# GEO Data Mining Identifies OLR1 as a Potential Biomarker in NSCLC Immunotherapy

**DOI:** 10.3389/fonc.2021.629333

**Published:** 2021-04-20

**Authors:** Bin Liu, Ziyu Wang, Meng Gu, Cong Zhao, Teng Ma, Jinghui Wang

**Affiliations:** ^1^ Department of Cellular and Molecular Biology, Beijing Chest Hospital, Capital Medical University, Beijing Tuberculosis and Thoracic Tumor Research Institute, Beijing, China; ^2^ Department of Medical Oncology, Beijing Chest Hospital, Capital Medical University, Beijing Tuberculosis and Thoracic Tumor Research Institute, Beijing, China

**Keywords:** non-small cell lung cancer, immunotherapy, tumor microenvironment, PD-L1, immune checkpoint

## Abstract

Non-small cell lung cancer (NSCLC) is the most common type of lung cancer. The tumor immune microenvironment (TME) in NSCLC is closely correlated to tumor initiation, progression, and prognosis. TME failure impedes the generation of an effective antitumor immune response. In this study, we attempted to explore TME and identify a potential biomarker for NSCLC immunotherapy. 48 potential immune-related genes were identified from 11 eligible Gene Expression Omnibus (GEO) data sets. We applied the CIBERSORT computational approach to quantify bulk gene expression profiles and thereby infer the proportions of 22 subsets of tumor-infiltrating immune cells (TICs); 16 kinds of TICs showed differential distributions between the tumor and control tissue samples. Multiple linear regression analysis was used to determine the correlation between TICs and 48 potential immune-related genes. Nine differential immune-related genes showed statistical significance. We analyzed the influence of nine differential immune-related genes on NSCLC immunotherapy, and OLR1 exhibited the strongest correlation with four well-recognized biomarkers (PD-L1, CD8A, GZMB, and NOS2) of immunotherapy. Differential expression of OLR1 showed its considerable potential to divide TICs distribution, as determined by non-linear dimensionality reduction analysis. In immunotherapy prediction analysis with the comparatively reliable tool TIDE, patients with higher OLR1 expression were predicted to have better immunotherapy outcomes, and OLR1 expression was potentially highly correlated with PD-L1 expression, the average of CD8A and CD8B, IFNG, and Merck18 expression, T cell dysfunction and exclusion potential, and other significant immunotherapy predictors. These findings contribute to the current understanding of TME with immunotherapy. OLR1 also shows potential as a predictor or a regulator in NSCLC immunotherapy.

## Introduction

Lung cancer is one of the most commonly occurring malignancies and is identified as leading cause of cancer-related deaths worldwide. Statistics on cancer reveal that in 2018, more than 2 million patients suffered from lung cancer worldwide, and lung cancer ranked first among all cancer types (1). In China, lung cancer has shown the highest incidence and mortality over the last decade and posed a significant threat to human health (2). Lung cancer comprises nearly 20% of all cancer deaths and its mortality may increase by approximately 40% by 2030 (3). Lung cancer has been subdivided based on pathological classification into two groups: small-cell lung cancer (SCLC) and Non-small cell lung cancer (NSCLC). NSCLC is further stratified into three subsets: adenocarcinoma (the most common type, which comprises 40% of all lung cancer types), squamous cell carcinoma (25%) and large cell carcinoma (about 10%) (4). 75% of NSCLC is diagnosed at the advanced stage, resulting in a five-year survival rate of less than 15% (5). In addition to surgery and chemoradiotherapy, immunotherapy shows promising potential in treatment of NSCLC.

Programmed death-1 (PD-1) and its ligand (PD-L1) antibody based Immunotherapies have recently shown significant advances in the treatment of NSCLC through enhancing the attack of the host immune system on malignant cells (6). However, a number of patients still fail to benefit from these immunotherapies possibly because of tumor immune microenvironment (TME) alterations (7). The tumor microenvironment includes cross-talks among cancer cells, endothelial cells, fibroblasts and immune cells. Previous studies have shown the potential associations between the microenvironment and the effects of immunotherapy (8). The tumor microenvironment presents physical, immunologic, and metabolic barriers to enduring immunotherapy responses, and the suppressive microenvironment of tumors remains one of the limiting factors for immunotherapies. In this study, we screened those genes that enriched in tumor tissue and closely related to the immune microenvironment in NSCLC. Targeting PD-L1, one of the most representative immunotherapy strategies, was studied in relation to the screened microenvironment genes. The response prediction markers of PD-L1 were used as standard and to study the predictive value of each gene as biomarker for host tumor immunity in NSCLC. We aimed to find a highly predictive PD-L1 blockade therapy biomarker for clinical use, or the potential targeting genes, to change the tumor microenvironment for enhanced immunotherapy effects.

## Materials and Methods

### Raw Data

Eligible data sets in GEO were selected according to the following criteria and 11 data sets were obtained as of May 2020 (including 268 cases of control samples, 601 cases of lung tumor samples) (9–18). Data sets details are listed in **Table 1**. The selection criteria are as follows: definite diagnosis with NSCLC; inclusion of control samples in the same data set; samples without any treatment before sequencing; gene expression data based on the Affymetrix platform. After normalization of mRNA data with the limma algorithm in R language, all data sets were merged into a new data set for downstream analysis.

**Table 1:** The detail of datasets using in this study.

Data Set	Country	Platforms	Diagnosis	Paired	Control	Tumor	Control after filter	Tumor after filter
**GSE18842**	Spain	GPL570	NSCLC	Part	45	46	45	45
**GSE101929**	USA	GPL570	NSCLC	Part	34	32	32	30
**GSE103888**	UK	GPL570	NSCLC	No	6	13	6	13
**GSE104636**	Switzerland	GPL6244	lung tumor	Yes	9	9	5	0
**GSE118370**	China	GPL570	LUAD	Yes	6	6	0	1
**GSE134381**	UK	GPL11532	LUSC/LUAD	Yes	37	37	22	22
**GSE19804**	China	GPL570	lung tumor	Yes	60	60	60	58
**GSE23361**	USA	GPL5188	NSCLC	part	7	5	0	1
**GSE30219**	France	GPL570	lung tumor	No	14	293	14	254
**GSE33532**	Germany	GPL570	NSCLC	Yes	20	20	20	19
**GSE43458**	USA	GPL6244	LUAD	part	30	80	18	49

NSCLC, there was clear statement that cancer tissues from NSCLC patients, but pathological classification not included in original article; Lung tumor, there was no clear statement about lung cancer subtype, but small cell lung cancer was excluded; LUAD, articles had a clear statement of lung adenocarcinoma. LUSC, articles had a clear statement of lung squamous cell carcinoma.

### Identification and Analysis of DEGs

Data were analyzed using the limma package in the R language. Details on cutoffs were as follows: Fold change>1 or <−1, and adj. P <0.05. Heatmaps and volcano plots were generated using the limma package and pheatmap respectively. Gene ontology (Go) and Kyoto Encyclopedia of Genes and Genomes (KEGG) analyses with “clusterProfiler” and “org.Hs.eg.db” (the same as below) were performed in R.

### Identification of Potential Immune-Related Genes and Analysis

In this study, the algorithm ESTIMATE outputs stromal, immune, and ESTIMATE scores by performing “limma” and “estimate” with merged data sets after normalization (19). The cutoffs for high or low scores were 50% higher or lower. The Venn diagram was drawn with the package “VennDiagram” and an online tool from the Bioinformatics & Systems Biology website. The intersection set of the results of the ESTIMATE scores were considered as control intersection genes and tumor intersect genes off the up-regulated and down-regulated genes respectively. In the subsequent step, the consistently intersecting set was excluded from those tumor intersect genes, and the remaining genes were considered as tumor specific intersect genes. The intersection set of the tumor specific intersect genes and DEGs were identified as potential immune-related genes and used for further analysis.

### Identification of Statistically TICs

The normalized data set was employed to estimate the TICs abundance profile by using the CIBERSORT computational method on all control and tumor samples (20). The resulting data set was filtered with a self-compiled script in Perl to exclude invalid data (detail of filtered data set is listed in **Table 1**). The landscape of TICs is shown in a barplot. A heatmap was generated with “pheatmap,” and a correlation heatmap was generated using “corrplot.” As the filtered data were subjected to a normality test based on skewness and kurtosis rather than its fit in a normal distribution, non-parametric tests were used. For the number of TICs in the control and tumor samples, the differential distributions were analyzed using the Wilcoxon rank-sum test.

### Multiple Linear Regression Analysis for Identifying Differential Immune-Related Genes From Potential Immune-Related Genes

The filtered data were used to analyze the effect of gene expression on the TICs distribution. A total of 16 TICs with different distributions were entered as dependent variable and potential immune-related genes entered as independent variables. Predictive factor analysis for TICs distribution was conducted *via* least-squares regression. With both accuracy and computational efficiency considered, adjusted P< 0.1 was considered significant in this part. We selected the significantly ones from 48 potential immune-related genes and identified those exhibiting the opposite regression trend in different tissue samples. Those selected genes would be labeled as differential immune-related genes.

### Gene Set Enrichment Analysis With Differential Immune-Related Genes

Gene set enrichment analysis (GSEA) was conducted on all differential immune-related genes on the GSEA portal (http://www.broad.mit.edu/GSEA/) with the following parameter settings: probe set collapse = false; phenotype = high vs. low; permutation: sample, permutations = 1000. The gene set size was 15 < n < 500. We manually discriminated pathways of interest in immune related pathways, microenvironment and metabolic pathways, and classic cancer pathways. The GSEA results were separately shown based on the pathway function.

### Non-Linear Dimensionality Reduction

In this study, we selected one differential immune-related gene that is most stable and highly correlated with other well-recognized immunotherapy signatures to perform downstream analysis, which was OLR1 (oxidized low density lipoprotein receptor 1). We determined whether OLR1 was a key gene for the microenvironment and could be a biomarker for immunotherapy. We first analyzed the influence of OLR1 expression to divide the differential immune microenvironment with non-linear dimensionality reduction. We used t-distributed stochastic neighbor embedding (t-SNE) to complete the dimensionality reduction. T-SNE, a non-linear dimensionality reduction technique, is particularly suitable for visualizing high-dimensional data sets. We reduced the dimensionality to two dimensions to reveal the significant difference between the 50% higher OLR1 expression tissue samples and the 50% lower OLR expression tissue samples. The T-SNE plot was completed using the R package “t-SNE.”

### Prediction of Benefits to Immune Checkpoint Blockade Therapy

We used TIDE, an online prediction tool to predict the responder rate in the samples with higher or lower OLR1 expression under immune checkpoint blockade therapy. TIDE is a novel computational framework that evaluates the potential of tumor immune escape, particularly for melanoma and NSCLC, on the basis of gene expression data (21). Owing to tool limitations, only the top or bottom 50 cases of OLR expression data were selected from the 284 samples. The result was reorganized into a figure with improved readability. We collected predictive indicators and calculated them to explore the details of the predicated therapeutic effect.

### Cell Culture

We used BEAS-2B (human normal lung epithelial cell, Cat. 3131C0001000200027), NCI-H460 (human large cell lung cancer cell, Cat. 3111C0001CCC000355), PLA-801D (lung giant cell carcinoma cell, Cat. 3142C0001000000356), A549 (human non-small cell lung cancer cell, Cat. 3111C0001CCC000002), HCC827 (human non-small cell lung cancer cell, Cat. 3111C0001CCC000478), NCI-H1299 (human non-small cell lung cancer cell, Cat. 3111C0001CCC000469), and NCI-H661 (human large cell lung cancer cell, Cat. 3111C0001CCC000357) to detect the basic expression of OLR1 in lung normal and tumor cell lines for verification of data mining results. They were purchased from Chinese National Infrastructure of Cell Line Resource and cultured in PRMI-1640 medium with 10% FBS.

### qPCR

RNA was isolated with TRIzol^®^ reagent (Cat.15596018, Thermo Fisher). Reverse-transcription of the RNA was performed with EasyScript First-Strand cDNA Synthesis SuperMix Kit (Cat. AE301-03, TransGen Biotech). The qPCR assay was performed in triplicate with PowerUp SYBE Green Master Mix Kit (Cat.A25741, Applied Biosystems) on an ABI StepOnePlus Real-time PCR system (ABI-7500, Applied Biosystems). The qPCR primer was following: OLR1-F:5′-ACTCTCCATGGTGGTGCCTGG-3′; OLR1-R:5′-GCTTGTTGCCGGGCTGAGATCT-3′; GAPDH-F:5′-GGACTCATGACCACAGTCCATGCC-3′; GAPDH-R:5′-TCAGGGATGACCTTGCCCACAG-3′.

### Statistical Methods

Microarray data analysis was performed using the R programming language. The normality of data distributions was assessed using the Shapiro-Wilk test. Data that were nor normally distributed were compared using the Wilcoxon rank-sum test run with GraphPad Prism. Multivariate analysis was performed using multiple linear regression in Eviews. P value < 0.05 was considered significant.

## Results

### 613 Differentially Expressed Genes Were Identified in 11 Data Sets

**Figure 1:**
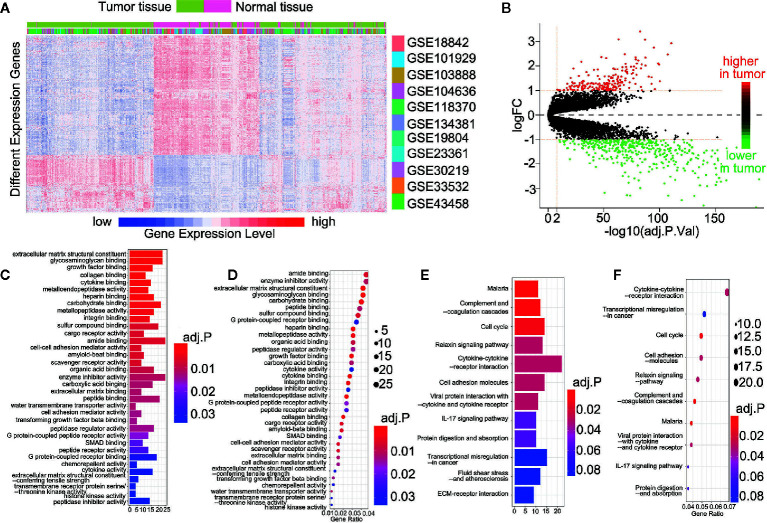
613 DEGs were identified in 11 datasets. **(A)** Heatmap showed the expression of genes between the tumor and control tissue samples; **(B)** Scatterplot showed the higher or lower DEGs between the tumor and control tissue samples; **(C)** The Go analyses of higher DEGs; **(D)** The KEGG analyses of higher DEGs; **(E)** The Go analyses of lower DEGs; **(F)** The KEGG analyses of lower DEGs.

For this study, 11 data sets were selected based on our screening criteria **(** **Table 1** **)**. After the data sets were merged and normalized, 601 NSCLC samples and 268 control samples were included. We defined statistical differential significance as P < 0.05 and fold change >10 between the tumor and control samples. A total of 613 differentially expressed genes (DEGs) were identified from 869 samples with 12596 gene expression data. Their gene symbols are listed based on their respective logFC (base 2 logarithm of fold change) values (**Supplementary Table 1**). The results revealed that 200 of the DEGs were highly expressed, whereas the remaining 413 DEGs were down-regulated in tumor tissues (**Figures 1A, B**). GO and KEGG pathway enrichment analyses of up-regulated or down-regulated DEGs were implemented separately. The up-regulated DEGs were enriched in the extracellular matrix structural constituent, glycosaminoglycan binding and growth factor binding (**Supplementary Table 2** and **Figure 1C**). The corresponding signaling pathways were the most enriched (**Supplementary Table 3** and **Figure 1D**). The down-regulated DEGs mainly involved the cytokine-cytokine receptor interaction, transcriptional miss-regulation in cancer, and cell cycle, as well as corresponding gene functions (**Figures 1E, F**).

### 166 Tumor-Specific Intersect Genes Were Identified Using ESTIMATE Scores, With 48 of Them Identified as Potential Immune-Related Genes by the Intersection With DEGs

**Figure 2:**
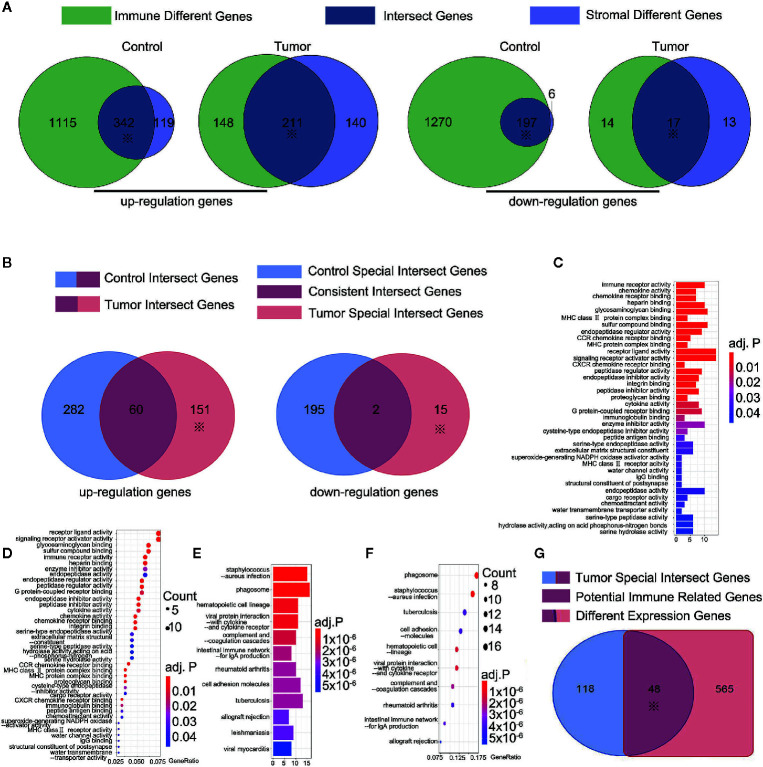
166 tumor-special intersect genes were identified and 48 of them identified as potential immune-related genes. **(A)** 461 (203) and 351 (30) up-regulated (down-regulated) genes were identified using ESTIMATE in the control and tumor samples. ※ mean this part would be used in following step; **(B)** 151 up-regulated and 15 down-regulated ESTIMATE score genes (total 166 tumor-special intersect genes) were specifically expressed in the tumor samples; **(C)** The Go analyses of 151 up-regulated tumor-special intersect genes; **(D)** The KEGG analyses of 151 up-regulated tumor-special intersect genes; **(E)**. The Go analyses of 15 down-regulated tumor-special intersect genes; **(F)** The KEGG analyses of 15 down-regulated tumor-special intersect genes; **(G)** 48 intersected genes, referred to potential immune-related genes, were identified between 613 DEGs and 166 tumor-special intersect genes.

ESTIMATE scores were calculated based on immune and stromal scores. We calculated the immune and stromal scores in the tumor and control samples, respectively. The samples were divided into the high-score and low-score groups by the median value of the immune score (or stromal score). For the immune score, 1,457 (1,276) and 359 (31) up-regulated (down-regulated) immune scores genes were identified in the control and tumor samples, respectively. For the stromal score, 461 (203) and 351 (30) up-regulated (down-regulated) genes were identified in the control and tumor samples, respectively (**Figure 2A**). The intersection represented the genes with the same trends of up-regulation or down-regulation of immune or stromal score genes in the tumor and control samples. In the control samples, we identified 342 up-regulated immune score genes and 197 down-regulated stromal score genes. Meanwhile, 211 up-regulation immune score genes and 17 down-regulated stromal score genes were found in the tumor samples (**Figure 2A**). A total of 553 up-regulated genes and 214 down-regulated genes were identified in the tumor and control samples (**Figure 2B**). They were regarded as important factors for ESTIMATE scores. Subsequently, 60 up-regulated and two down-regulated genes were duplicates, exhibiting a similar trend of immune or stromal score genes were similar in the tumor and control samples (**Figure 2B**). A concerning finding of this study is that 151 up-regulated and 15 down-regulated ESTIMATE score genes were specifically expressed in the tumor samples **(** **Supplementary Table 4** and **Figure 2B** with *). Thus, the 166 tumor specific intersect genes were selected for GO enrichment and KEGG pathway analysis. They were enriched in pathways including, immune receptor activity, chemokine activity and receptor binding, receptor ligand activity, glycosaminoglycan binding, phagosome, and lgA immune network (**Supplementary Tables 5** and **6**, **Figures 2C–F**). We then found the intersection of 166 tumor specific intersect genes and 613 DEGs and identified 48 intersected genes (**Supplementary Table 7** and **Figure 2G**).The 48 genes were identified as potential immune-related genes. Potential immune-related genes have significant differences in expression and specific immune effects in the tumor samples.

### 16 Kinds of TICs Showed Differences in Distribution Between the Tumor and Control Samples

**Figure 3:**
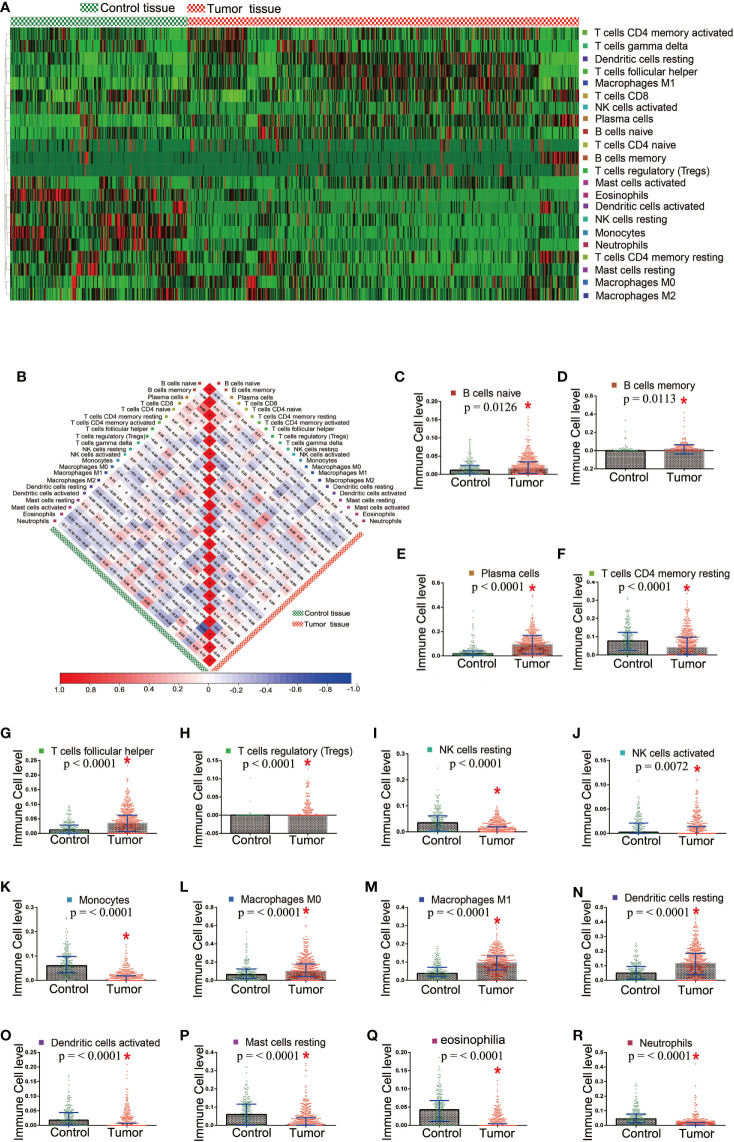
Different distribution of TICs between the tumor and control tissue samples. **(A)** Heatmap showed the distribution state in tumor and control tissue samples; **(B)** The correlation among TICs in the tumor and control tissue samples; **(C–R)** 16 TICs which showed significant difference in the distribution between the tumor and control tissue samples.

The proportion of tumor-infiltrating immune subsets was analyzed using CIBERSORT in R. A total of 22 TICs profile types in the tumor and control samples were to be constructed. A landscape is presented for preliminary subjective judgment of TICs distributions (**Supplementary Figure 1**). We found that TICs distribution varied based on the kind of TICs. Some of TICs had more immune cells in tumor tissues, whereas others showed the opposite (**Figure 3A**). No significant correlation was found between them with regard to numerical values in both the tumor samples and the control samples (**Figure 3B**). The Mann-Whitney rank-sum test was used to calculate differences in TICs distribution. A significant difference in the distribution of 16 kinds of TICs was found between the tumor and control samples **(** **Supplementary Table 8** and **Figures 3C–R**).

### 9 Differentially Expressed Immune-Related Genes Showed Opposite Statistically Significant Regression Trends of TICs Distribution Between the Tumor and Control Samples

**Figure 4:**
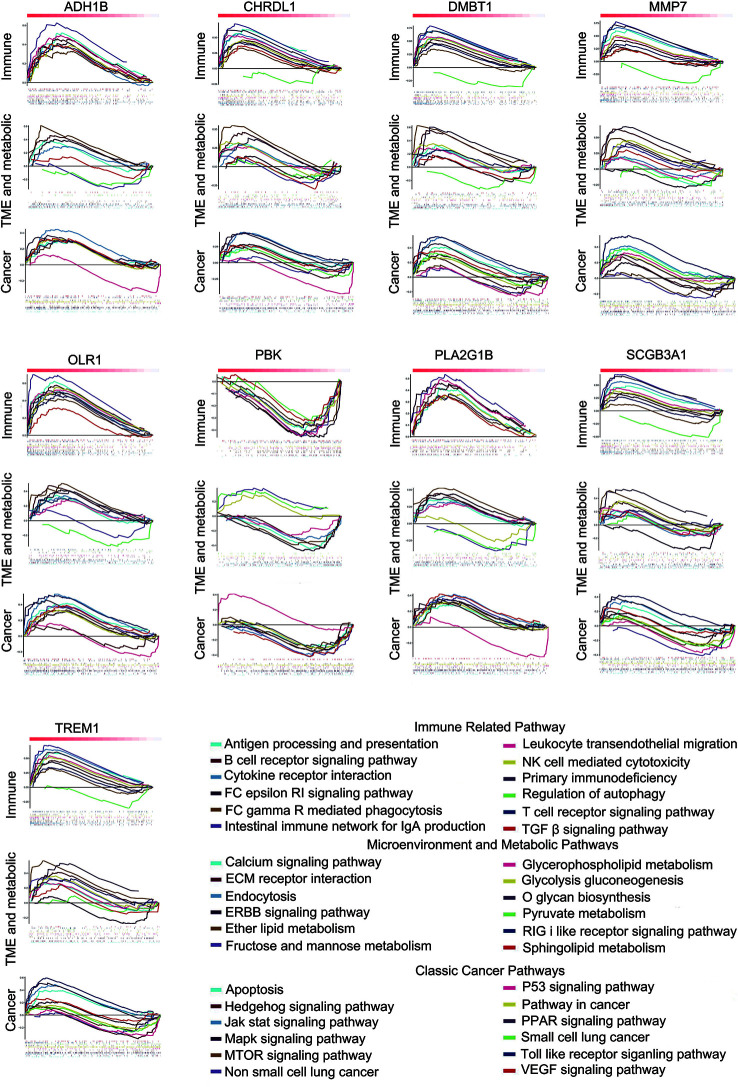
Some pathways could be activated or repressed by each of the 9 differential immune-related genes.

We used multiple linear regression with the least square method to calculate the most important genes from 48 potential immune-related genes for differential the distribution of TICs. The control and tumor samples were separated as independent data sets. For an enhanced screen effect, P<0.1 was the level of significance selected. A total of 24 intersected genes were statistically significant both in the tumor and control samples; 16 of these genes showed similar regression trends for some kinds of TICs in both samples (**Supplementary Table 9**), and 9 other genes exhibited the opposite regression trend in different tissue samples (**Supplementary Table 10**). OLR1 showed the same regression trend for resting natural killer (NK) cells and eosinophils; however, it exhibited the opposite regression trend for resting mast cell. Thus, OLR1 was counted separately. The genes showing opposite regression trends between the tumor and control samples were referred to as differential immune-related genes in accordance with the purpose of the study. Subsequently, 9 differential immune-related genes were identified: ADH1B, CHRDL1, DMBT1, MMP, OLR1, PBK, PLA2G1B, SCGB3A1, and TREM1. The potential functions of these genes were analyzed by GSEA. We classified some important pathways into three categories, based on their function for clarity. Some pathways could be activated/repressed by each of the 9 differential immune-related genes (**Figure 4**).

### OLR1, One of the Differential Immune-Related Genes, Showed Significant Correlations With Four Known Immunotherapy Biomarkers

**Figure 5:**
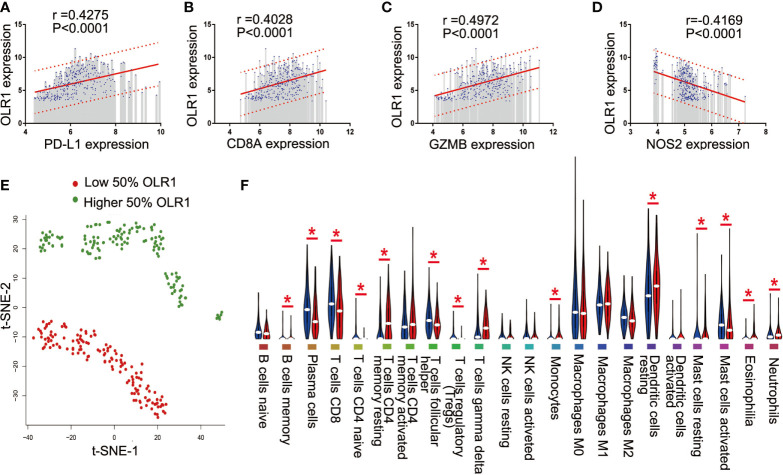
OLR1 showed moderately strong correlations with 4 known immunotherapy biomarkers and its expression marked the TICs distribution. **(A–D)** OLR1 exhibited the highest correlation with PD-L1, CD8A, GZMB, and NOS2; **(E)** OLR1 expression could divide overall status of TICs distribution; **(F)** Violin plot showed the ratio differentiation of 22 kinds of TICs between higher 50% OLR1 expression tumor samples and lower 50% OLR1 expression tumor samples.

Since the data were from 11 data sets merged with different platforms, and some data were lost during merging, we selected only 4 well-recognized biomarkers in the data set with gene expression data: PD-L1, CD8A, GZMB, and NOS2. To reduce potential bias and achieve improved effectiveness, we used only part of the data for downstream analysis. The 284 tumor samples in GSE101929 and GSE30219 with clinical data were used to calculate separately the correlation between 9 differential immune-related genes and 4 known biomarkers. Consequently, OLR1 exhibited the highest correlation with PD-L1, CD8A, GZMB, and NOS2 (**Supplementary Table 11** and **Figures 5A–D**), and each correlation was significant (r>0.4, P<0.0001, moderate intensity). Chi-square test was used to detect whether different expression level of known biomarker was followed by changing of OLR1 expression level. We can found more positive expression for immunotherapy in higher OLR1 group patients (**Supplementary Table 12**). This result suggests the important role of OLR1 in the TME with a changing TICs distribution for immunotherapy prediction.

### OLR1 Expression Marked the TICs Distribution

We ran t-SNE to determine the overall distribution of TICs with different levels of OLR1 expression. The OLR1 expression could effectively distinguish the distribution of immune cells (**Figure 5E**). A violin plot of 22 immune cell types shows 14 kinds of immune cells with significantly different distributions between the higher and lower OLR1 expression samples (**Figure 5F**).

### OLR1 Affected the Prediction of Clinical Benefits to Immunotherapy in NSCLC Patients

**Figure 6:**
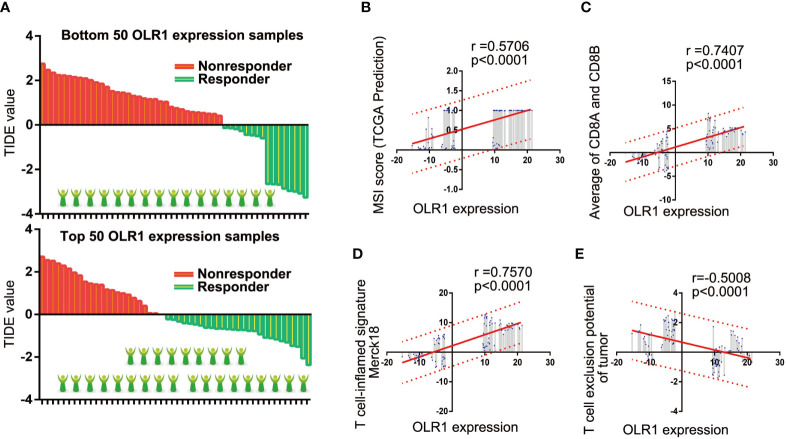
OLR1 may affect the clinical benefits to immunotherapy in NSCLC patients. **(A)** The comparison of responder number in top or bottom OLR1 expression samples. **(B–E)** OLR1 has strong positive correlation with some indicators of TIDE prediction.

We selected the 50 samples with the highest or lowest OLR1 expression levels from the 284 tumor samples and predicted the responder rate of the immune checkpoint blockade therapy in each group. Among the 50 samples with the lowest OLR1 expression levels, 16 cases were predicted to respond to immune checkpoint blockade therapy. They were also predicted to benefit from immunotherapy. The responder number was 27 in the top 50 OLR1 expression samples. The responder rate was significantly higher in the high OLR1 expression group (**Figure 6A**). We also calculated all indicators of TIDE prediction. OLR1 expression and MSI score exhibited a significant positive correlation. The T cell exclusion potential of the tumor was predicted to be negatively correlated with OLR1 expression. Specifically, a strong positive correlation (r>0.7) was observed between OLR1 expression and the T cell-inflamed signature (Merck18), the average of CD8A and CD8B, both of which were important indicators of immunotherapy (**Supplementary Table 13** and **Figures 6B–E**).

### Verification of OLR1 Expression in Lung Normal and Tumor Cell Lines

**Figure 7:**
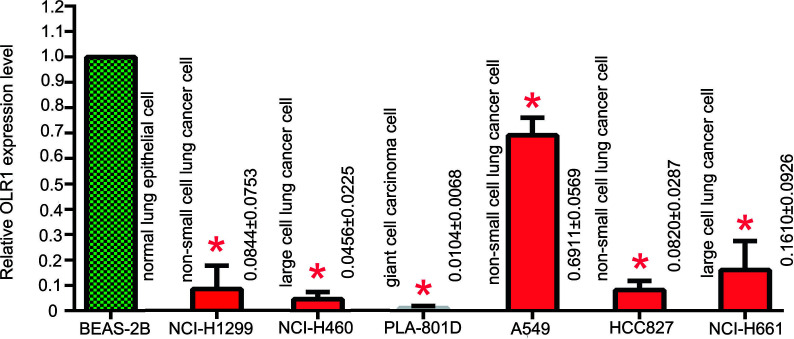
The basic expression level of OLR1 in normal and part NSCLC cell lines.

BEAS-2B was human normal lung epithelial cell. It was used as control cell line comparison with other NSCLC cell lines. The result of qPCR analysis showed a significant reduction in the expression of OLR1 in the most NSCLC cell lines (**Figure 7**). The validation result of cell lines had the same trend as data mining in OLR1 expression.

## Discussion

In the current study based on GEO data mining, we identified NSCLC microenvironment-related key genes, which show potential as biomarkers for immunotherapy. During the analysis, significant expressions of DEGs were identified between the control and tumor tissue samples; meanwhile, tumor-specific intersect genes also showed significant difference between the immune and stromal components of the tumor samples; further intersection of DEGs and tumor-specific intersect genes identified potential immune-related genes, both of which were considered different gene expression and significantly affected the ESTIMATE TME scores; finally differential immune-related genes exhibited a significant and opposite correlation with the differential distribution of TICs between the tumor and control samples. OLR1 was considered as a novel potential predictor to immunotherapy of NSCLC.

DEG analysis has been demonstrated as a canonical way to identify novel biomarker genes or novel therapeutic target genes between the normal and tumor samples. In this study, DEG acted as a signature of potential immune-related factors. The screening accuracy of DEGs may be improved using two methods (22). A large sample is expected to improve the screening yield, and analysis with perspective-taking may help improve accuracy based on multiple bioinformatics methods. Other studies randomly chose data sets; in this study, we filtered all GEO data sets by using the selection criteria (see Methods), ultimately including 11 data sets in our analysis cohort. There were 601 tumor samples and 268 control samples across 7 countries and 11 research groups. As our selection criteria, all the 11 data sets should include tumor and normal tissue data for better comparability and higher quality of homogenization data. All data sets were also based on the Affymetrix platform for less data loss in the normalization process. A total of 613 DEGs were identified based on the mRNA array data. These results may be representative and provide an insight in the differences in gene expression in NSCLC. The 613 DEGs were the limited scope for screening in downstream analysis.

The TME is rather complex and largely varies from the microenvironment of normal tissue (7). The TME has been implicated in cancer initiation, development, and treatment resistance. Except for cancer cells, the stromal tumor microenvironment consists of stromal cells and immune cells. The immune cells include T cells, B cells, NK cells, and so on. Stromal cells are the main non-tumor components of the tumor microenvironment (23). Thus, the ESTIMATE algorithm consists of a non-immune “stromal score” parameter and an “immune score” parameter. In this study, the immune and stromal scores, which were determined using ESTIMATE, were used to analyze the infiltration levels of the immune and stromal cells in the tumor and control samples. We focused on genes that specifically regulated the stromal and immune cells in the tumor samples, referred to as the tumor specific intersect gene. We found 166 tumor specific intersect genes in our cohort. They may exhibit cancer-specific effects and show potential to highly regulate the development of the TME in the tumor tissue samples. To determine the type of tumor specific intersect genes with the potential to regulate and control expression; thus the intersecting of the tumor specific intersect genes and DEGs was identified as potential immune-related genes.

Immune cells in TME are the most important defense to eliminate damaged or cancerous cells (23). An established view is that the spatial distribution and the location of immune cells are critical in the immune-oncology field (24). In this study, we tried to identify the differential distributions of immune cells, particularly for TICs. CIBERSORT estimate the abundances of cell types in a mixed cell population based on gene expression data. The CIBERSORT method identified 16 kinds of TICs in our cohort, which were significantly different between the tumor and control samples and not correlated among them. They may each play an independent role in the tumor environment. We explored the correlation between the TICs and 48 potential immune-related genes; the results of multiple non-linear regression analysis indicated that 9 potential immune-related genes with opposite significant correlation between the control and tumor tissue samples. Referred to as differential immune-related genes, those 9 potential immune-related genes, may have tumor tissue-specific functions in the regulation of the immune environment. We evaluated their potential influence on the biological function by using GSEA, and the results showed their potential to activate or inactivate multiple types of signaling pathways, including the immune pathway, microenvironment, and metabolic pathway, and some canonical cancer pathways. The pathways are widely recognized as cancer regulatory mechanisms.

Though much effort has been on identifying genes as biomarkers for immunotherapy, little progress has been made. We thus determined the predicted values for differential immune-related genes in NSCLC. We selected well-recognized biomarkers with gene expression data in our data set (25). PD-L1, also named as B7-H1 or CD274, is the first ligand of PD-1 discovered and widely expressed. It is the main factor responsible for promoting tumor immune evasion (26), and has been a major clinical target for immunotherapy in NSCLC. CD8A is one of the hallmarks of CD8+ T-cell activation signature genes, as well as GZMB (27). NOS2 is the classically activated macrophage transcript (28). They are widely used in multiple studies to predict the efficacy of immunotherapy. In this study, we calculate the relationship between differential immune-related genes and 4 well-recognized immunotherapy biomarkers. OLR1 showed the best correlation with those existing biomarkers and thus was selected to explore its specific relationship with TME. PD-L1 is the most important clinical predictors of immunotherapy and its expression level in tumor tissue determines whether to apply immunotherapy. The NSCLC patient with higher PD-L1 would be believed get better clinical benefit from PD-1/PD-L1 antibody therapy. In this study, there were numerically positive correlations between PD-L1 and OLR1. It may imply that OLR1 could be used as an auxiliary diagnostic marker for immunotherapy. CD8+ activation is the signature event of anti-cancer effect for immune-checkpoint inhibitors. The positive numerically correlation between OLR1 and CD8+ could provide further support for OLR1 plays as a biomarker of immunotherapy. Notably, all analyses were based on gene expression data and all positive or negative correlations just showed statistically significant. However, they have not been assessed in either clinical or experimental models.

Moreover, t-SNE analysis showed a significant difference in the distribution of immune cells with 50% high or low OLR1 expression tumor samples. This finding suggests that OLR1 can distinguish the state of TME by influencing the distribution of immune cells. Fourteen kinds of immune cells changed in OLR1 expression level, and the ratios (low or high) of four well-recognized immunotherapy biomarkers were significantly different between the low and high OLR1 expression groups. Furthermore, we found an interesting result when we compared TILs distribution between “normal vs. tumor” and “lower OLR1 expression vs. higher OLR1 expression” (**Figures 3C–R**, and **Figure 5F**). There were 16 kinds of TILs with different distribution between normal tissue and tumor tissue and 14 kinds of different distribution TILs between lower OLR1 expression tissue and higher OLR1 expression tissue. Ten kinds of TILs were identical in both comparisons. A term TILs (B cells memory, plasma cells, T cells follicular helper and T cells regulatory) have more distribution in tumor tissue (compared with normal tissue) and lower OLR1 expression tissue (compared with higher OLR1 expression tissue), and another term (T cells CD4 memory resting, monocytes, eosinophilia and neutrophils) showed the opposite distribution. It indicates that lower OLR1 expression has close relationship with tumor microenvironment. OLR1 also named LOX1. It was first identified as a scavenger receptor for oxLDL in bovine aortic endothelias cells (29). It is also expressed in macrophages, vascular smooth muscle cells, platelets and tumor cells (30). Its overexpression enhances the migration in breast *via* NF-κB (31). The transcription of OLR1 could be regulated by multiple transcriptional factors, and its activation depended on a wide range of stimuli indicative of dyslipidemia, inflammation and damage initiates several signaling cascades including MAPKs, other protein kinases as well as transcription factors NF-κB and AP-1 (32).

The predictive values of biomarkers should support clinical values. We used TIDE to detect the predictive effect of OLR1 on NSCLC immunotherapy. TIDE is a gene expression biomarker for predicting clinical response to immune checkpoint blockade. Melanoma and NSCLC data are included in the data sets. Given the limitation of the website, we chose 100 tumor samples (top 50 and bottom 50 samples of OLR1 expression in the last cohort) to run the TIDE prediction process. Results showed that the prediction response rate in the top 50 OLR1 expression group was 54% (27/50), which was significantly higher than that in the bottom 50 OLR1 expression group (16/50). In the comparison of other predictive indicators of TIDE, OLR1 expression showed statistical relevance. The results suggested that OLR1 could be used as a biomarker for immunotherapy in NSCLC. As the main disadvantage of data mining study, there are number of limitations of the work. In this study, all analyses used the same data set and all data was from gene expression micro-assay. The single source of data might affect the reliability of the results. Meanwhile, data are derived from multiple countries in this study. Though we set explicit criteria before normalization of data sets, it is inevitable that data missing happens, or some data from different sources may interfere and cancel each other. Therefore, the results might have been biased to a certain degree. In addition, the algorithms are also immature, though we used the well-accepted ones, such as limma, ESTIMATE or CIBERSORT. At last, TIDE, a novel online tool to predict immunotherapy outcome with gene expression data, is used as the last analyses of OLR1 in this research. It is likely that there is no enough evidence for authenticity and reliability of the TIDE prediction results. Those data mining results may change with different data processing or novel algorithm. However, it also provided some clues now for our study and we are trying to validated it experimentally for more evidence. In verification the basic gene expression of OLR1 in multiple NSCLC cell lines, the result showed the significant reduction of OLR1 level in most NSCLC cells than normal lung cell line.

This study explores TME with bioinformatics analysis of public gene expression data sets in NSCLC. Multi-omics data mining shows its reliability in screening meaningful genes in NSCLC with public data sets. The principal findings of this research are that OLR1 played a key role in TME and could predict or potentially be regulated for NSCLC immunotherapy. OLR1 expression was correlated with some well-recognized biomarkers of immunotherapy, including PD-L1, CD8A, GZMB, NOS2, and other predictors. OLR1 expression could divide the differential TICs distribution, and those patients with higher OLR1 expression were predicted to obtain more benefits from immunotherapy in NSCLC. Given the limitations in time and technology, the regulatory role and molecular mechanism of OLR1 were not investigated in depth; regardless, the properties of OLR1 indicate its potential value in NSCLC immunotherapy. We intend to continue this study in future research and explore more clinical data.

## Data Availability Statement

The original contributions presented in the study are included in the article/**Supplementary Material**. Further inquiries can be directed to the corresponding authors.

## Author Contributions

Concept and design: TM and JW. Administrative support: MG and ZW. Collection and assembly of data: BL and CZ. Data analysis and interpretation: BL, TM, and JW. Manuscript writing: all authors. Final approval of manuscript: all authors. All authors contributed to the article and approved the submitted version.

## Conflict of Interest

The authors declare that the research was conducted in the absence of any commercial or financial relationships that could be construed as a potential conflict of interest.

## References

[1] Siegel RL Miller KD Jemal A. Cancer statistics, 2020. CA: Cancer J Clin (2020) 70(1):7–30. 10.3322/caac.21590 31912902

[2] Chen W Zheng R Baade PD Zhang S Zeng H Bray F. Cancer statistics in China, 2015. CA: Cancer J Clin (2016) 66(2):115–32. 10.3322/caac.21338 26808342

[3] Alberg AJ Brock MV Samet JM. Epidemiology of lung cancer: looking to the future. J Clin Oncol Off J Am Soc Clin Oncol (2005) 23(14):3175–85. 10.1200/JCO.2005.10.462 15886304

[4] Duruisseaux M Esteller M. Lung cancer epigenetics: From knowledge to applications. Semin Cancer Biol (2018) 51:116–28. 10.1016/j.semcancer.2017.09.005 28919484

[5] Duma N Santana-Davila R Molina JR. Non-Small Cell Lung Cancer: Epidemiology, Screening, Diagnosis, and Treatment. Mayo Clin Proc (2019) 94(8):1623–40. 10.1016/j.mayocp.2019.01.013 31378236

[6] Osmani L Askin F Gabrielson E Li QK. Current WHO guidelines and the critical role of immunohistochemical markers in the subclassification of non-small cell lung carcinoma (NSCLC): Moving from targeted therapy to immunotherapy. Semin Cancer Biol (2018) 52(Pt 1):103–9. 10.1016/j.semcancer.2017.11.019 PMC5970946 29183778

[7] Osipov A Saung MT Zheng L Murphy AG. Small molecule immunomodulation: the tumor microenvironment and overcoming immune escape. J Immunother Cancer (2019) 7(1):224. 10.1186/s40425-019-0667-0 31439034 PMC6704558

[8] Musetti S Huang L. Nanoparticle-Mediated Remodeling of the Tumor Microenvironment to Enhance Immunotherapy. ACS Nano (2018) 12(12):11740–55. 10.1021/acsnano.8b05893 30508378

[9] Calverley DC Phang TL Choudhury QG Gao B Oton AB Weyant MJ. Significant downregulation of platelet gene expression in metastatic lung cancer. Clin Trans Sci (2010) 3(5):227–32. 10.1111/j.1752-8062.2010.00226.x PMC3427741 21500395

[10] Fregni G Quinodoz M Moller E Vuille J Galland S Fusco C. Reciprocal modulation of mesenchymal stem cells and tumor cells promotes lung cancer metastasis. EBioMedicine (2018) 29:128–45. 10.1016/j.ebiom.2018.02.017 PMC5925622 29503225

[11] Hoang LT Domingo-Sabugo C Starren ES Willis-Owen SAG Morris-Rosendahl DJ Nicholson AG. Metabolomic, transcriptomic and genetic integrative analysis reveals important roles of adenosine diphosphate in haemostasis and platelet activation in non-small-cell lung cancer. Mol Oncol (2019) 13(11):2406–21. 10.1002/1878-0261.12568 PMC6822241 31461552

[12] Kabbout M Garcia MM Fujimoto J Liu DD Woods D Chow CW. ETS2 mediated tumor suppressive function and MET oncogene inhibition in human non-small cell lung cancer. Clin Cancer Res Off J Am Assoc Cancer Res (2013) 19(13):3383–95. 10.1158/1078-0432.CCR-13-0341 PMC3846434 23659968

[13] Kuo CS Liu CY Pavlidis S Lo YL Wang YW Chen CH. Unique Immune Gene Expression Patterns in Bronchoalveolar Lavage and Tumor Adjacent Non-Neoplastic Lung Tissue in Non-Small Cell Lung Cancer. Front Immunol (2018) 9:232. 10.3389/fimmu.2018.00232 29483918 PMC5816075

[14] Lu TP Hsiao CK Lai LC Tsai MH Hsu CP Lee JM. Identification of regulatory SNPs associated with genetic modifications in lung adenocarcinoma. BMC Res Notes (2015) 8:92. 10.1186/s13104-015-1053-8 25889623 PMC4384239

[15] Mitchell KA Zingone A Toulabi L Boeckelman J Ryan BM. Comparative Transcriptome Profiling Reveals Coding and Noncoding RNA Differences in NSCLC from African Americans and European Americans. Clin Cancer Res Off J Am Assoc Cancer Res (2017) 23(23):7412–25. 10.1158/1078-0432.CCR-17-0527 PMC8171584 29196495

[16] Rousseaux S Debernardi A Jacquiau B Vitte AL Vesin A Nagy-Mignotte H. Ectopic activation of germline and placental genes identifies aggressive metastasis-prone lung cancers. Sci Trans Med (2013) 5(186):186ra66. 10.1126/scitranslmed.3005723 PMC4818008 23698379

[17] Sanchez-Palencia A Gomez-Morales M Gomez-Capilla JA Pedraza V Boyero L Rosell R. Gene expression profiling reveals novel biomarkers in nonsmall cell lung cancer. Int J Cancer (2011) 129(2):355–64. 10.1002/ijc.25704 20878980

[18] Xu L Lu C Huang Y Zhou J Wang X Liu C. SPINK1 promotes cell growth and metastasis of lung adenocarcinoma and acts as a novel prognostic biomarker. BMB Rep (2018) 51(12):648–53. 10.5483/BMBRep.2018.51.12.205 PMC6330943 30545439

[19] Yoshihara K Shahmoradgoli M Martinez E Vegesna R Kim H Torres-Garcia W. Inferring tumour purity and stromal and immune cell admixture from expression data. Nat Commun (2013) 4:2612. 10.1038/ncomms3612 24113773 PMC3826632

[20] Newman AM Steen CB Liu CL Gentles AJ Chaudhuri AA Scherer F. Determining cell type abundance and expression from bulk tissues with digital cytometry. Nat Biotechnol (2019) 37(7):773–82. 10.1038/s41587-019-0114-2 PMC6610714 31061481

[21] Jiang P Gu S Pan D Fu J Sahu A Hu X. Signatures of T cell dysfunction and exclusion predict cancer immunotherapy response. Nat Med (2018) 24(10):1550–8. 10.1038/s41591-018-0136-1 PMC6487502 30127393

[22] Vougas K Sakellaropoulos T Kotsinas A Foukas GP Ntargaras A Koinis F. Machine learning and data mining frameworks for predicting drug response in cancer: An overview and a novel in silico screening process based on association rule mining. Pharmacol Ther (2019) 203:107395. 10.1016/j.pharmthera.2019.107395 31374225

[23] Quail DF Joyce JA. Microenvironmental regulation of tumor progression and metastasis. Nat Med (2013) 19(11):1423–37. 10.1038/nm.3394 PMC3954707 24202395

[24] Holmgaard RB Schaer DA Li Y Castaneda SP Murphy MY Xu X. Targeting the TGFbeta pathway with galunisertib, a TGFbetaRI small molecule inhibitor, promotes anti-tumor immunity leading to durable, complete responses, as monotherapy and in combination with checkpoint blockade. J Immunother Cancer (2018) 6(1):47. 10.1186/s40425-018-0356-4 29866156 PMC5987416

[25] Camidge DR Doebele RC Kerr KM. Comparing and contrasting predictive biomarkers for immunotherapy and targeted therapy of NSCLC. Nat Rev Clin Oncol (2019) 16(6):341–55. 10.1038/s41571-019-0173-9 30718843

[26] Jiang Y Zhan H. Communication between EMT and PD-L1 signaling: New insights into tumor immune evasion. Cancer Lett (2020) 468:72–81. 10.1016/j.canlet.2019.10.013 31605776

[27] Oja AE Piet B van der Zwan D Blaauwgeers H Mensink M de Kivit S. Functional Heterogeneity of CD4(+) Tumor-Infiltrating Lymphocytes With a Resident Memory Phenotype in NSCLC. Front Immunol (2018) 9:2654. 10.3389/fimmu.2018.02654 30505306 PMC6250821

[28] Kahn DA Archer DC Gold DP Kelly CJ. Adjuvant immunotherapy is dependent on inducible nitric oxide synthase. J Exp Med (2001) 193(11):1261–8. 10.1084/jem.193.11.1261 PMC2193375 11390433

[29] Sawamura T Kume N Aoyama T Moriwaki H Hoshikawa H Aiba Y. An endothelial receptor for oxidized low-density lipoprotein. Nature (1997) 386(6620):73–7. 10.1038/386073a0 9052782

[30] Hirsch HA Iliopoulos D Joshi A Zhang Y Jaeger SA Bulyk M. A transcriptional signature and common gene networks link cancer with lipid metabolism and diverse human diseases. Cancer Cell (2010) 17(4):348–61. 10.1016/j.ccr.2010.01.022 PMC2854678 20385360

[31] Khaidakov M Mitra S Kang BY Wang X Kadlubar S Novelli G. Oxidized LDL receptor 1 (OLR1) as a possible link between obesity, dyslipidemia and cancer. PloS One (2011) 6(5):e20277. 10.1371/journal.pone.0020277 21637860 PMC3102697

[32] Li D Saldeen T Romeo F Mehta JL. Oxidized LDL upregulates angiotensin II type 1 receptor expression in cultured human coronary artery endothelial cells: the potential role of transcription factor NF-kappaB. Circulation (2000) 102(16):1970–6. 10.1161/01.CIR.102.16.1970 11034947

