# Supplementary material for: GEO Data Mining Identifies OLR1 as a Potential Biomarker in NSCLC Immunotherapy
Source: Front Oncol. 2021 Apr 20;11:629333. doi: 10.3389/fonc.2021.629333 (PMC8095246; doi:10.3389/fonc.2021.629333)
Supplement: Supplementary file 1 [file DataSheet_1.docx]

**Supplementary Information**


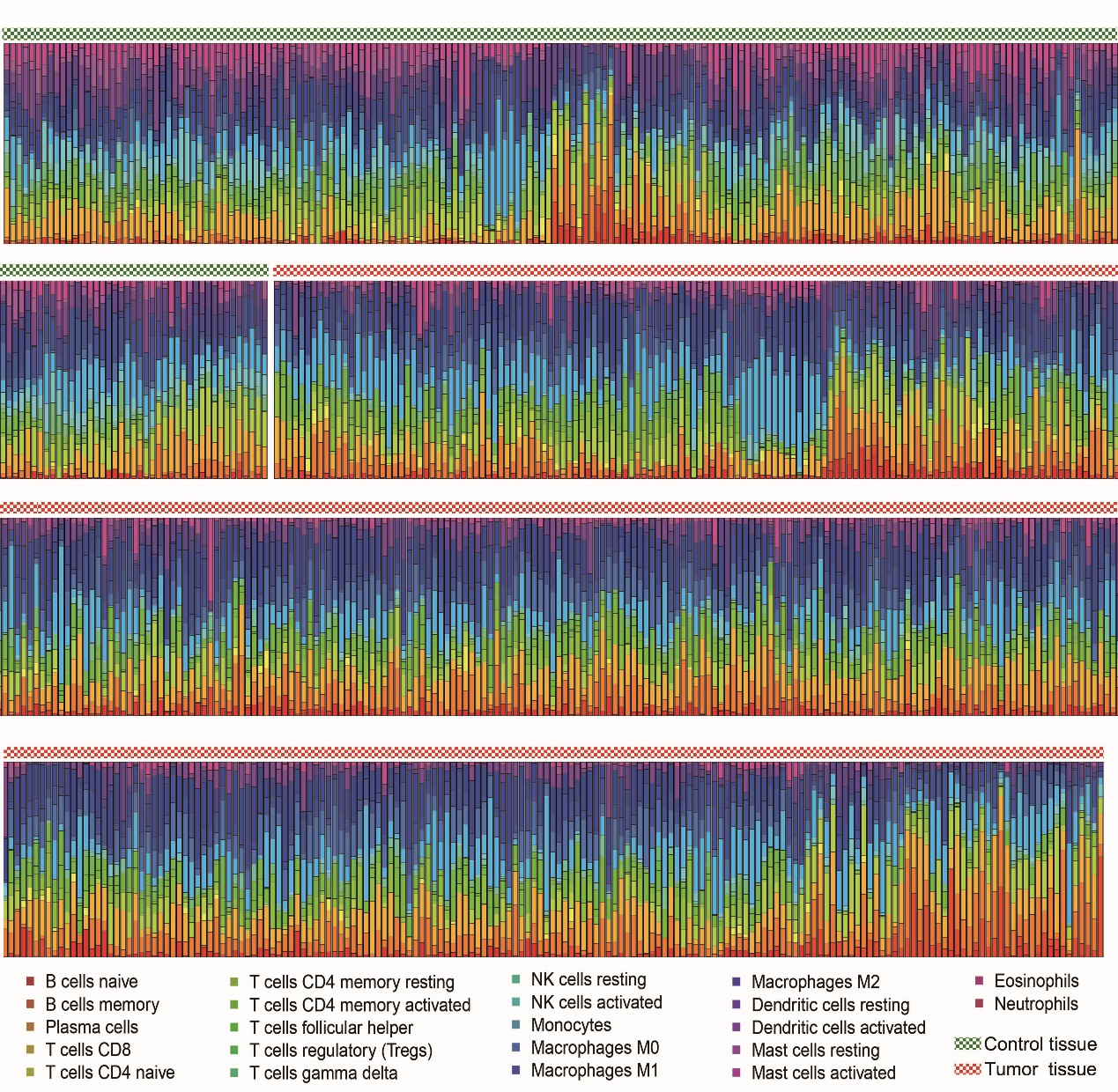


**Supplementary Figure 1. landscape of TICs distributions.**

Supplementary Table1. A total of 613 DEGs were identified between the tumor and control tissue samples.

| Up-regulated DEGs in tumor | | | | | |
| --- | --- | --- | --- | --- | --- |
| ID | logFC | **ID** | logFC | **ID** | logFC |
| MMP12 | 3.402148 | **MMP1** | 3.077643 | **GREM1** | 3.068735 |
| SPP1 | 2.923115 | **COL10A1** | 2.744673 | **TMPRSS4** | 2.476083 |
| CTHRC1 | 2.468622 | **CXCL13** | 2.468622 | **COL11A1** | 2.349276 |
| ANLN | 2.337143 | **GJB2** | 2.337143 | **DLGAP5** | 2.259287 |
| TPX2 | 2.217831 | **SPINK1** | 2.217831 | **TOP2A** | 2.098434 |
| RRM2 | 2.098223 | **CDC20** | 2.098223 | **GINS1** | 2.063207 |
| UBE2T | 2.059407 | **SULF1** | 2.059407 | **MELK** | 1.976437 |
| CEP55 | 1.964253 | **CDH3** | 1.964253 | **CRABP2** | 1.949942 |
| THBS2 | 1.904784 | **CCNB1** | 1.904784 | **PBK** | 1.894184 |
| HS6ST2 | 1.889293 | **AKR1B10** | 1.889293 | **NUF2** | 1.866235 |
| TTK | 1.862718 | **CDKN3** | 1.862718 | **CST1** | 1.851902 |
| ADAMDEC1 | 1.839453 | **KIF20A** | 1.839453 | **UBE2C** | 1.809387 |
| KIF4A | 1.789915 | **PSAT1** | 1.789915 | **CP** | 1.74575 |
| KIF11 | 1.730275 | **HMGB3** | 1.730275 | **PRC1** | 1.694214 |
| HMMR | 1.675351 | **ANKRD22** | 1.675351 | **ZWINT** | 1.633847 |
| SCG5 | 1.621508 | **FOXM1** | 1.621508 | **GPR87** | 1.590013 |
| GCNT3 | 1.588985 | **DSP** | 1.588985 | **NDC80** | 1.574313 |
| KIF14 | 1.573775 | **KIAA0101** | 1.573775 | **NCAPG** | 1.561868 |
| FAP | 1.542767 | **COL1A1** | 1.542767 | **CCNA2** | 1.511596 |
| NEK2 | 1.51154 | **S100A2** | 1.51154 | **CDC6** | 1.501341 |
| COL3A1 | 1.491527 | **CDCA7** | 1.491527 | **MMP9** | 1.45733 |
| KIF2C | 1.44955 | **EZH2** | 1.44955 | **PTTG1** | 1.42797 |
| MKI67 | 1.408025 | **MMP13** | 1.408025 | **CDK1** | 1.391969 |
| SLC2A1 | 1.387177 | **HJURP** | 1.387177 | **CENPE** | 1.378096 |
| OCIAD2 | 1.372286 | **COL5A2** | 1.372286 | **NMU** | 1.365209 |
| C15orf48 | 1.354866 | **MAD2L1** | 1.354866 | **TK1** | 1.345863 |
| ASPM | 1.341276 | **LGSN** | 1.341276 | **MMP11** | 1.328758 |
| GPT2 | 1.322527 | **CENPF** | 1.322527 | **STIL** | 1.317938 |
| RMI2 | 1.309712 | **IGFBP3** | 1.309712 | **RAD51AP1** | 1.30116 |
| KIF15 | 1.300485 | **CXCL14** | 1.300485 | **TRIP13** | 1.297088 |
| GPX2 | 1.293329 | **SLC7A5** | 1.293329 | **NQO1** | 1.287781 |
| AFAP1-AS1 | 1.287331 | **CDCA5** | 1.287331 | **PCP4** | 1.282639 |
| TRIM59 | 1.28002 | **MCM2** | 1.28002 | **DEPDC1** | 1.278249 |
| PLAU | 1.275971 | **IGF2BP3** | 1.275971 | **DEPDC1B** | 1.268904 |
| KRT15 | 1.268119 | **BIRC5** | 1.268119 | **PYCR1** | 1.264718 |
| AURKA | 1.263878 | **FERMT1** | 1.263878 | **MXRA5** | 1.259539 |
| STEAP1 | 1.253771 | **DSG2** | 1.253771 | **CENPK** | 1.243662 |
| MDK | 1.243099 | **CDC45** | 1.243099 | **PLOD2** | 1.237641 |
| ADAM12 | 1.222699 | **MAP7D2** | 1.222699 | **MND1** | 1.209524 |
| FAM83D | 1.204783 | **HIST1H2BD** | 1.204783 | **RPL39L** | 1.202225 |
| RGS17 | 1.201545 | **UGT8** | 1.201545 | **EXO1** | 1.19058 |
| CYP24A1 | 1.190208 | **SLC12A8** | 1.190208 | **SLC7A11** | 1.186989 |
| IGSF9 | 1.180331 | **CEACAM5** | 1.180331 | **TYMS** | 1.173598 |
| COMP | 1.173571 | **SPC25** | 1.173571 | **RFC4** | 1.16855 |
| CDCA8 | 1.167896 | **MMP7** | 1.158262 | **SPOCK1** | 1.157939 |
| TMPRSS11E | 1.157939 | **PLEK2** | 1.151898 | **NCAPH** | 1.147429 |
| PAFAH1B3 | 1.147429 | **SHCBP1** | 1.140392 | **EGLN3** | 1.140145 |
| CDCA3 | 1.140145 | **ARNTL2** | 1.134831 | **SLC6A8** | 1.13341 |
| HMGB3P1 | 1.126148 | **PTGFRN** | 1.126148 | **SIX1** | 1.124783 |
| COL5A1 | 1.124595 | **SFN** | 1.124595 | **PRAME** | 1.115908 |
| MYBL2 | 1.115572 | **SPAG4** | 1.115572 | **SRD5A1** | 1.108061 |
| PPAT | 1.104916 | **CLDN10** | 1.104916 | **SGPP2** | 1.099344 |
| CHEK1 | 1.098931 | **HIST1H2BH** | 1.092277 | **DTL** | 1.091755 |
| LAD1 | 1.091755 | **CKAP2L** | 1.091275 | **POLE2** | 1.082715 |
| METTL7B | 1.082715 | **OIP5** | 1.07499 | **XDH** | 1.068471 |
| TFAP2A | 1.068471 | **DNAJC12** | 1.068301 | **TNFRSF21** | 1.063202 |
| THY1 | 1.063202 | **CCNE2** | 1.059812 | **FAM83A** | 1.056876 |
| FUT2 | 1.056876 | **ASF1B** | 1.051403 | **GGCT** | 1.048437 |
| KDELR3 | 1.048437 | **SERPINB5** | 1.041999 | **CENPU** | 1.039999 |
| FANCI | 1.039999 | **NCAPG2** | 1.036534 | **SPRR1B** | 1.032524 |
| GALNT7 | 1.032524 | **MCM4** | 1.031515 | **WISP1** | 1.029469 |
| GALNT14 | 1.029469 | **APOBEC3B** | 1.023373 | **HORMAD1** | 1.022314 |
| ABCA12 | 1.022314 | **TCN1** | 1.019261 | **SUGCT** | 1.014562 |
| ETV4 | 1.014562 | **KISS1R** | 1.011095 | **KNTC1** | 1.008484 |
| FDCSP | 1.008484 | **GJB6** | 1.00681 | **ECT2** | 1.00634 |
| PDIA4 | 1.00634 | **MFAP2** | 1.004517 | **KRT6B** | 1.004271 |
| ATAD2 | 1.004271 | **CKS1B** | 1.001165 |  |  |
| Down-regulated DEGs in tumor | | | | | |
| ID | logFC | **ID** | logFC | **ID** | logFC |
| AGER | -3.78876 | **TMEM100** | -3.69195 | **CLDN18** | -3.47686 |
| FCN3 | -3.30629 | **GKN2** | -3.29381 | **WIF1** | -3.28965 |
| SFTPC | -3.27546 | **SCGB1A1** | -3.17716 | **CPB2** | -2.98776 |
| FABP4 | -2.90735 | **SLC6A4** | -2.87286 | **TNNC1** | -2.85599 |
| MAMDC2 | -2.79864 | **CYP4B1** | -2.76597 | **FHL1** | -2.73438 |
| FAM107A | -2.72518 | **PEBP4** | -2.66932 | **EDNRB** | -2.66815 |
| ADH1B | -2.65065 | **GPM6A** | -2.62294 | **MT1M** | -2.58972 |
| NCKAP5 | -2.57705 | **CA4** | -2.56619 | **SDPR** | -2.5555 |
| MCEMP1 | -2.52332 | **AQP4** | -2.51437 | **SOSTDC1** | -2.49475 |
| EMCN | -2.44956 | **TCF21** | -2.43329 | **ACADL** | -2.37927 |
| FOSB | -2.3469 | **CHRDL1** | -2.3468 | **C2orf40** | -2.31766 |
| IGSF10 | -2.28905 | **LYVE1** | -2.26985 | **ANKRD29** | -2.26044 |
| MFAP4 | -2.21681 | **CAV1** | -2.1792 | **SERTM1** | -2.17398 |
| FAM150B | -2.16639 | **SCN7A** | -2.15138 | **NOSTRIN** | -2.14759 |
| FAM189A2 | -2.1341 | **PGC** | -2.12477 | **ADIRF** | -2.11763 |
| CLIC5 | -2.09964 | **LRRN3** | -2.07839 | **PLA2G1B** | -2.06842 |
| OGN | -2.06166 | **ZBTB16** | -2.03482 | **VIPR1** | -2.02131 |
| HHIP | -2.0175 | **CDH5** | -2.01748 | **STX11** | -2.01086 |
| MS4A15 | -2.00955 | **GPIHBP1** | -2.00302 | **CACNA2D2** | -1.98283 |
| TGFBR3 | -1.97791 | **SFTPD** | -1.97002 | **HSD17B6** | -1.96138 |
| PPBP | -1.96 | **JAM2** | -1.94403 | **LPL** | -1.94166 |
| GDF10 | -1.93008 | **HIGD1B** | -1.92916 | **HBB** | -1.92776 |
| KL | -1.91899 | **CD36** | -1.91267 | **MARCO** | -1.90316 |
| IL33 | -1.87332 | **CALCRL** | -1.87188 | **GNG11** | -1.86962 |
| MME | -1.85644 | **AOC3** | -1.85373 | **IL6** | -1.85119 |
| PTPRB | -1.84996 | **RTKN2** | -1.84377 | **GPX3** | -1.82001 |
| SLIT2 | -1.81629 | **BCHE** | -1.81233 | **STXBP6** | -1.80994 |
| CFD | -1.80966 | **SCGB3A2** | -1.80771 | **RAMP3** | -1.80227 |
| LAMP3 | -1.79549 | **ANXA3** | -1.79234 | **C20orf85** | -1.78533 |
| MMRN1 | -1.78474 | **PDK4** | -1.78423 | **ADAMTS8** | -1.78398 |
| ZNF385B | -1.77669 | **KANK3** | -1.77612 | **TPPP3** | -1.77056 |
| FOXF1 | -1.75745 | **SUSD2** | -1.75718 | **CCBE1** | -1.745 |
| KLF4 | -1.74306 | **AQP1** | -1.73383 | **ADRB2** | -1.72639 |
| FXYD1 | -1.72174 | **HPGD** | -1.71897 | **TSPAN7** | -1.71328 |
| S1PR1 | -1.6964 | **LRRC36** | -1.68839 | **OLR1** | -1.6846 |
| ARHGEF26 | -1.68321 | **ACKR1** | -1.68302 | **CYYR1** | -1.6635 |
| FGFBP2 | -1.65367 | **DACH1** | -1.65052 | **EPAS1** | -1.64743 |
| SPOCK2 | -1.64399 | **SOX7** | -1.63755 | **SGCG** | -1.63193 |
| SELE | -1.62972 | **RAMP2** | -1.62644 | **FIBIN** | -1.6237 |
| ABCA3 | -1.61861 | **CSRNP1** | -1.61341 | **GIMAP6** | -1.60922 |
| SCN4B | -1.6071 | **TRHDE** | -1.60342 | **SEMA3G** | -1.58783 |
| ESAM | -1.58451 | **FBLN5** | -1.58272 | **GPR146** | -1.57604 |
| ACVRL1 | -1.571 | **EMP2** | -1.56753 | **FLRT3** | -1.55998 |
| PPP1R14A | -1.55895 | **RXFP1** | -1.55831 | **NDNF** | -1.55738 |
| CLEC14A | -1.55595 | **CLDN5** | -1.55463 | **TEK** | -1.54154 |
| S100A12 | -1.53392 | **SLCO2A1** | -1.53179 | **SLC46A2** | -1.52908 |
| ITM2A | -1.52513 | **HYAL1** | -1.52205 | **ANGPTL1** | -1.52022 |
| SLC1A1 | -1.51895 | **PCOLCE2** | -1.51669 | **KLRF1** | -1.51384 |
| GPM6B | -1.50793 | **FILIP1** | -1.50575 | **SRPX** | -1.50107 |
| VGLL3 | -1.49839 | **SELP** | -1.49228 | **WISP2** | -1.4913 |
| CLIC3 | -1.48193 | **GIMAP8** | -1.48133 | **GRIA1** | -1.47953 |
| KCNK3 | -1.47949 | **ADAMTS1** | -1.47497 | **C7** | -1.47188 |
| CLEC1A | -1.47011 | **LIMCH1** | -1.46675 | **CHI3L2** | -1.46236 |
| CD93 | -1.46041 | **ABCA6** | -1.45885 | **LINC00312** | -1.45684 |
| ASPA | -1.45651 | **VEPH1** | -1.45333 | **AGTR1** | -1.4525 |
| CXCR2 | -1.45236 | **MYCT1** | -1.45209 | **PLEKHH2** | -1.45018 |
| ZBED2 | -1.44897 | **FRMD3** | -1.44723 | **LRRC32** | -1.44321 |
| ROBO4 | -1.43852 | **FHL5** | -1.43164 | **RGCC** | -1.4305 |
| SOX17 | -1.42795 | **THBD** | -1.42684 | **GHR** | -1.42184 |
| FOLR1 | -1.41887 | **ANGPT1** | -1.4145 | **RHOJ** | -1.41011 |
| NRN1 | -1.40533 | **KCNT2** | -1.3965 | **PPP1R15A** | -1.3954 |
| SEMA5A | -1.39505 | **PLLP** | -1.39032 | **C1QTNF7** | -1.38864 |
| SLC14A1 | -1.38221 | **ADRB1** | -1.38021 | **LIMS2** | -1.37642 |
| FCN1 | -1.37398 | **SCARA5** | -1.37254 | **CYP2B7P** | -1.3719 |
| GIMAP7 | -1.36972 | **BMP2** | -1.36614 | **PECAM1** | -1.36492 |
| HSPB8 | -1.36331 | **CRYAB** | -1.36253 | **SFTPB** | -1.36019 |
| NEXN | -1.35979 | **P2RY14** | -1.35669 | **C4BPA** | -1.35463 |
| VSIG4 | -1.35321 | **CD52** | -1.35312 | **ITIH5** | -1.35104 |
| CCDC68 | -1.34961 | **BTNL9** | -1.34855 | **F8** | -1.34787 |
| ABCA8 | -1.34781 | **GRK5** | -1.34562 | **CPA3** | -1.34455 |
| ICAM2 | -1.34001 | **HLF** | -1.33579 | **CD69** | -1.33442 |
| LIFR | -1.33361 | **PLAC8** | -1.33212 | **SCEL** | -1.33158 |
| C14orf132 | -1.32968 | **SASH1** | -1.3292 | **PPARG** | -1.32893 |
| SYNM | -1.32494 | **SOCS2** | -1.32354 | **AOX1** | -1.32233 |
| BMP5 | -1.31581 | **FGD5** | -1.31298 | **AKAP12** | -1.31229 |
| FGR | -1.30882 | **CXCL3** | -1.30481 | **GLIPR2** | -1.30354 |
| FAM216B | -1.30245 | **TMEM47** | -1.30054 | **IL18R1** | -1.30038 |
| DPEP2 | -1.29845 | **SLC19A3** | -1.2971 | **TMEM178A** | -1.29485 |
| FOS | -1.29389 | **NTN4** | -1.2882 | **SPARCL1** | -1.28612 |
| CA2 | -1.28581 | **PALMD** | -1.28408 | **LDB2** | -1.28204 |
| S100A3 | -1.27255 | **FAM46B** | -1.27021 | **RAI2** | -1.26573 |
| WASF3 | -1.26398 | **CSF3** | -1.26317 | **ITGA8** | -1.25802 |
| SELENBP1 | -1.25624 | **WFDC1** | -1.25494 | **RNF182** | -1.25294 |
| ST6GALNAC3 | -1.25177 | **CDO1** | -1.25171 | **MMRN2** | -1.24952 |
| CAB39L | -1.24614 | **NPNT** | -1.24515 | **DAPK2** | -1.24245 |
| VWF | -1.24203 | **MS4A2** | -1.24147 | **LGI3** | -1.23844 |
| ERG | -1.23517 | **TMEM139** | -1.23504 | **PLA1A** | -1.23447 |
| CNTN6 | -1.23033 | **ZFP36** | -1.22857 | **HPGDS** | -1.22769 |
| GIMAP1 | -1.22746 | **OLFML1** | -1.22742 | **ARRB1** | -1.22151 |
| HEG1 | -1.21975 | **FXYD6** | -1.21386 | **RAPGEF4** | -1.21245 |
| EML1 | -1.21105 | **AGR3** | -1.21009 | **MYADM** | -1.21007 |
| EDN1 | -1.20785 | **ARHGAP6** | -1.2077 | **CORO2B** | -1.20638 |
| TCEAL2 | -1.20464 | **TMOD1** | -1.20407 | **COX7A1** | -1.19907 |
| SLPI | -1.19721 | **AGTR2** | -1.18991 | **TMEM88** | -1.18938 |
| FBP1 | -1.1864 | **SLC39A8** | -1.18457 | **ABCG2** | -1.18319 |
| NR4A2 | -1.18222 | **C1orf162** | -1.18152 | **PIP5K1B** | -1.1795 |
| PAPSS2 | -1.17547 | **PTGDS** | -1.17533 | **ADAMTSL3** | -1.17426 |
| C9orf24 | -1.17164 | **ICAM4** | -1.17146 | **PTPRM** | -1.16991 |
| DPYSL2 | -1.1683 | **LHFP** | -1.16358 | **NPR1** | -1.16242 |
| DYNLRB2 | -1.16051 | **CAMK2N1** | -1.1604 | **DMBT1** | -1.1598 |
| FPR2 | -1.15887 | **CXCL2** | -1.15884 | **CTNNAL1** | -1.15819 |
| CPED1 | -1.15643 | **ZBBX** | -1.14967 | **IL7R** | -1.14961 |
| ABI3BP | -1.14928 | **DUOX1** | -1.1474 | **RERG** | -1.14619 |
| PROK2 | -1.14524 | **VSIG2** | -1.14477 | **WFS1** | -1.14434 |
| CBX7 | -1.14336 | **PROS1** | -1.14264 | **DENND3** | -1.14106 |
| PTRF | -1.13883 | **SEMA6A** | -1.1379 | **RASL12** | -1.13707 |
| HSPB6 | -1.13616 | **CYBRD1** | -1.13518 | **GMFG** | -1.13445 |
| CCL23 | -1.12213 | **TRPC6** | -1.12207 | **CX3CR1** | -1.12132 |
| ADCY4 | -1.11852 | **RBMS3** | -1.11819 | **GPRC5A** | -1.11756 |
| KIAA1324L | -1.11702 | **PCDH17** | -1.11692 | **PDZD2** | -1.11679 |
| TOX2 | -1.11612 | **MYOCD** | -1.11386 | **ABLIM3** | -1.11362 |
| PDE2A | -1.11335 | **SLC6A14** | -1.11154 | **TIE1** | -1.11129 |
| GLDN | -1.11116 | **MAL** | -1.10768 | **GSTM5** | -1.10693 |
| GIMAP4 | -1.10546 | **RRAS** | -1.10156 | **KLF2** | -1.10121 |
| SCGB3A1 | -1.10096 | **GPC3** | -1.10063 | **ALOX5AP** | -1.09833 |
| MNDA | -1.0978 | **NEDD4L** | -1.09737 | **CASQ2** | -1.09639 |
| SBSPON | -1.0942 | **SYNC** | -1.09038 | **MYH10** | -1.087 |
| DPT | -1.0866 | **FMO3** | -1.08408 | **ITLN1** | -1.0839 |
| SH3BP5 | -1.0825 | **TREM1** | -1.08083 | **IL18RAP** | -1.08013 |
| GNLY | -1.0796 | **FRY** | -1.07912 | **STARD8** | -1.07737 |
| TGFBR2 | -1.07726 | **KLF6** | -1.07393 | **TMEM74B** | -1.07276 |
| PLSCR4 | -1.07029 | **KDR** | -1.06973 | **TNS1** | -1.06773 |
| C16orf89 | -1.06685 | **CCDC102B** | -1.06643 | **REEP1** | -1.06561 |
| PTGS2 | -1.06281 | **CCDC69** | -1.06136 | **ARHGEF6** | -1.06069 |
| ID4 | -1.05889 | **PRICKLE2** | -1.05854 | **ST6GALNAC5** | -1.0579 |
| RASSF2 | -1.0567 | **TM6SF1** | -1.05461 | **AQP9** | -1.05307 |
| TIMP3 | -1.05226 | **CAPN3** | -1.05171 | **C5AR1** | -1.05056 |
| GYPC | -1.04883 | **CFL2** | -1.04823 | **LRRN4** | -1.04748 |
| GPA33 | -1.04101 | **S100A4** | -1.04009 | **APOLD1** | -1.04 |
| CD34 | -1.03904 | **SEMA3E** | -1.03864 | **NAPSA** | -1.03532 |
| MYOC | -1.03375 | **PTPN21** | -1.03319 | **CASKIN2** | -1.03234 |
| MEIS1 | -1.03203 | **DLC1** | -1.02915 | **DNASE1L3** | -1.02896 |
| FERMT2 | -1.02845 | **PODXL** | -1.02126 | **PTX3** | -1.0201 |
| APOL3 | -1.0144 | **RGN** | -1.01406 | **SYNE1** | -1.01029 |
| SECISBP2L | -1.00984 | **FAT4** | -1.00962 | **SH2D3C** | -1.00947 |
| PKIG | -1.00944 | **MMP28** | -1.0089 | **FLI1** | -1.00627 |
| C8B | -1.00577 | **RSPH1** | -1.00568 | **PDE8B** | -1.00136 |
| GGTLC1 | -1.00087 | **EFEMP1** | -1.00068 |  |  |

Supplementary Table 2. The Go analyses of DEGs.

| ID | Description | GeneRatio | BgRatio | p.adjust | qvalue |
| --- | --- | --- | --- | --- | --- |
| GO:0005201 | Extracellular matrix structural constituent | 23/547 | 163/17696 | 8.73E-07 | 7.83E-07 |
| GO:0005539 | Glycosaminoglycan binding | 23/547 | 229/17696 | 0.000264 | 0.000237 |
| GO:0019838 | Growth factor binding | 16/547 | 137/17696 | 0.001075 | 0.000963 |
| GO:0005518 | Collagen binding | 11/547 | 67/17696 | 0.001075 | 0.000963 |
| GO:0019955 | Cytokine binding | 15/547 | 128/17696 | 0.001419 | 0.001272 |
| GO:0004222 | Metalloendopeptidase activity | 13/547 | 103/17696 | 0.002052 | 0.001839 |
| GO:0008201 | Heparin binding | 17/547 | 169/17696 | 0.002052 | 0.001839 |
| GO:0030246 | Carbohydrate binding | 22/547 | 271/17696 | 0.003469 | 0.00311 |
| GO:0008237 | Metallopeptidase activity | 17/547 | 181/17696 | 0.003855 | 0.003456 |
| GO:0005178 | Integrin binding | 14/547 | 132/17696 | 0.004288 | 0.003843 |
| GO:1901681 | Sulfur compound binding | 20/547 | 250/17696 | 0.006717 | 0.006021 |
| GO:0038024 | Cargo receptor activity | 10/547 | 76/17696 | 0.006717 | 0.006021 |
| GO:0033218 | Amide binding | 25/547 | 356/17696 | 0.006717 | 0.006021 |
| GO:0098632 | Cell-cell adhesion mediator activity | 8/547 | 50/17696 | 0.006717 | 0.006021 |
| GO:0001540 | Amyloid-beta binding | 10/547 | 78/17696 | 0.006717 | 0.006021 |
| GO:0005044 | Scavenger receptor activity | 8/547 | 51/17696 | 0.007043 | 0.006313 |
| GO:0043177 | Organic acid binding | 17/547 | 205/17696 | 0.009415 | 0.00844 |
| GO:0004857 | Enzyme inhibitor activity | 25/547 | 375/17696 | 0.011191 | 0.010032 |
| GO:0031406 | Carboxylic acid binding | 16/547 | 193/17696 | 0.012019 | 0.010774 |
| GO:0050840 | Extracellular matrix binding | 8/547 | 57/17696 | 0.012019 | 0.010774 |

Supplementary Table 3. The KEGG analyses of DEGs.

| ID | Description | GeneRatio | BgRatio | p.adjust | qvalue |
| --- | --- | --- | --- | --- | --- |
| hsa05144 | Malaria | 11/285 | 50/8048 | 0.000266 | 0.000244 |
| hsa04610 | Complement and coagulation cascades | 12/285 | 85/8048 | 0.005407 | 0.004972 |
| hsa04110 | Cell cycle | 14/285 | 124/8048 | 0.010415 | 0.009578 |
| hsa04926 | Relaxin signaling pathway | 13/285 | 129/8048 | 0.029885 | 0.027481 |
| hsa04060 | Cytokine-cytokine receptor interaction | 22/285 | 294/8048 | 0.029885 | 0.027481 |
| hsa04514 | Cell adhesion molecules | 14/285 | 148/8048 | 0.029885 | 0.027481 |
| hsa04061 | Viral protein interaction with cytokine and cytokine receptor | 11/285 | 100/8048 | 0.029885 | 0.027481 |
| hsa04657 | IL-17 signaling pathway | 10/285 | 94/8048 | 0.055982 | 0.05148 |
| hsa04974 | Protein digestion and absorption | 10/285 | 95/8048 | 0.055982 | 0.05148 |
| hsa05202 | Transcriptional misregulation in cancer | 15/285 | 192/8048 | 0.084627 | 0.077821 |
| hsa05418 | Fluid shear stress and atherosclerosis | 12/285 | 139/8048 | 0.084627 | 0.077821 |
| hsa04512 | ECM-receptor interaction | 9/285 | 88/8048 | 0.084627 | 0.077821 |
| hsa04115 | p53 signaling pathway | 8/285 | 73/8048 | 0.084627 | 0.077821 |
| hsa04670 | Leukocyte transendothelial migration | 10/285 | 113/8048 | 0.126763 | 0.116569 |
| hsa04614 | Renin-angiotensin system | 4/285 | 23/8048 | 0.143605 | 0.132057 |
| hsa04933 | AGE-RAGE signaling pathway in diabetic complications | 9/285 | 100/8048 | 0.147413 | 0.135559 |
| hsa04923 | Regulation of lipolysis in adipocytes | 6/285 | 56/8048 | 0.217639 | 0.200137 |
| hsa00480 | Glutathione metabolism | 6/285 | 57/8048 | 0.223116 | 0.205174 |
| hsa04062 | Chemokine signaling pathway | 13/285 | 189/8048 | 0.235037 | 0.216137 |
| hsa04668 | TNF signaling pathway | 9/285 | 112/8048 | 0.236402 | 0.217392 |

Supplementary Table 4. The list of 166 tumor-special intersect genes.

| DPT | SERPING1 | SFRP4 | PLN | RASSF2 | CD14 |
| --- | --- | --- | --- | --- | --- |
| IL10RA | ARHGEF6 | ENTPD1 | MFAP4 | C3AR1 | IL7R |
| SFRP2 | C3 | C1S | FGL2 | CCDC69 | MS4A6A |
| CYP1B1 | HLA-DMB | NEXN | CSF1R | ITK | C1QB |
| CD2 | BIRC3 | SLAMF8 | FCER1G | RAB31 | BTN3A3 |
| HLA-DMA | LAX1 | TYROBP | THEMIS2 | CD27 | CCL21 |
| LAPTM5 | MZB1 | CD86 | C1orf162 | NCF4 | GMFG |
| CCL2 | IL2RG | CCL5 | CCL18 | CORO1A | EMP3 |
| HLA-DRA | SLA | RARRES1 | SLAMF7 | CLEC5A | FPR3 |
| SASH3 | CD52 | PECAM1 | FYB | C7 | RARRES3 |
| PLEK | GIMAP4 | CCR7 | SRGN | TLR8 | HLA-DPA1 |
| NAPSA | OLR1 | LYVE1 | RASGRP1 | NDNF | NCF2 |
| CHRDL1 | GZMB | SLC34A2 | ICAM1 | PTPRC | LCP1 |
| TFEC | ABCA6 | CEACAM6 | SFTPB | CHI3L1 | CXCL13 |
| GLIPR1 | SLC6A14 | TREM1 | AQP9 | DMBT1 | GPNMB |
| FCN3 | AIM2 | ADH1B | CXCL17 | LTF | IGSF6 |
| STEAP4 | FDCSP | CXCL11 | MSR1 | MS4A1 | CXCL10 |
| PEBP4 | FBP1 | MUC1 | C16orf89 | PLA2G1B | AQP4 |
| CP | S100A8 | MSLN | ABI3BP | PGC | PIGR |
| SPINK1 | OGDHL | GNG4 | INA | RAB3B | CLGN |
| BEX1 | TUBB2B | CHGB | MRAP2 | NEFL | PCSK1 |
| PBK | BEX2 |  |  |  |  |

Supplementary Table 5. The GO analyses of 166 tumor-special intersect genes.

| ID | Description | GeneRatio | BgRatio | p.adjust | qvalue |
| --- | --- | --- | --- | --- | --- |
| GO:0140375 | Immune receptor activity | 10/152 | 128/17696 | 3.11E-05 | 2.54E-05 |
| GO:0008009 | Chemokine activity | 7/152 | 49/17696 | 3.11E-05 | 2.54E-05 |
| GO:0042379 | Chemokine receptor binding | 7/152 | 66/17696 | 0.000165 | 0.000134 |
| GO:0008201 | Heparin binding | 10/152 | 169/17696 | 0.000165 | 0.000134 |
| GO:0005539 | Glycosaminoglycan binding | 11/152 | 229/17696 | 0.000306 | 0.00025 |
| GO:0023026 | MHC class II protein complex binding | 4/152 | 16/17696 | 0.000477 | 0.00039 |
| GO:1901681 | Sulfur compound binding | 11/152 | 250/17696 | 0.000502 | 0.00041 |
| GO:0061135 | Endopeptidase regulator activity | 9/152 | 182/17696 | 0.001143 | 0.000934 |
| GO:0048020 | CCR chemokine receptor binding | 5/152 | 43/17696 | 0.001173 | 0.000958 |
| GO:0023023 | MHC protein complex binding | 4/152 | 25/17696 | 0.001875 | 0.001532 |
| GO:0048018 | Receptor ligand activity | 14/152 | 482/17696 | 0.002138 | 0.001747 |
| GO:0030546 | Signaling receptor activator activity | 14/152 | 487/17696 | 0.002187 | 0.001787 |
| GO:0045236 | CXCR chemokine receptor binding | 3/152 | 11/17696 | 0.002444 | 0.001997 |
| GO:0061134 | Peptidase regulator activity | 9/152 | 219/17696 | 0.002734 | 0.002233 |
| GO:0004866 | Endopeptidase inhibitor activity | 8/152 | 175/17696 | 0.002943 | 0.002404 |
| GO:0005178 | Integrin binding | 7/152 | 132/17696 | 0.002943 | 0.002404 |
| GO:0030414 | Peptidase inhibitor activity | 8/152 | 182/17696 | 0.003425 | 0.002798 |
| GO:0043394 | Proteoglycan binding | 4/152 | 36/17696 | 0.004507 | 0.003682 |
| GO:0005125 | Cytokine activity | 8/152 | 220/17696 | 0.010923 | 0.008923 |
| GO:0001664 | G protein-coupled receptor binding | 9/152 | 280/17696 | 0.011653 | 0.009519 |

Supplementary Table 6. The KEGG analyses of 166 tumor-special intersect genes.

| ID | Description | GeneRatio | BgRatio | p.adjust | qvalue |
| --- | --- | --- | --- | --- | --- |
| hsa05150 | Staphylococcus aureus infection | 15/93 | 96/8048 | 3.01E-11 | 2.45E-11 |
| hsa04145 | Phagosome | 16/93 | 152/8048 | 1.17E-09 | 9.47E-10 |
| hsa04640 | Hematopoietic cell lineage | 11/93 | 99/8048 | 7.36E-07 | 5.99E-07 |
| hsa04061 | Viral protein interaction with cytokine and cytokine receptor | 11/93 | 100/8048 | 7.36E-07 | 5.99E-07 |
| hsa04610 | Complement and coagulation cascades | 10/93 | 85/8048 | 1.44E-06 | 1.17E-06 |
| hsa04672 | Intestinal immune network for IgA production | 8/93 | 49/8048 | 2.15E-06 | 1.75E-06 |
| hsa05323 | Rheumatoid arthritis | 10/93 | 93/8048 | 2.45E-06 | 1.99E-06 |
| hsa04514 | Cell adhesion molecules | 12/93 | 148/8048 | 2.48E-06 | 2.01E-06 |
| hsa05152 | Tuberculosis | 13/93 | 180/8048 | 2.48E-06 | 2.01E-06 |
| hsa05330 | Allograft rejection | 7/93 | 38/8048 | 3.44E-06 | 2.79E-06 |
| hsa05140 | Leishmaniasis | 9/93 | 77/8048 | 3.44E-06 | 2.79E-06 |
| hsa05416 | Viral myocarditis | 8/93 | 60/8048 | 5.50E-06 | 4.47E-06 |
| hsa05332 | Graft-versus-host disease | 7/93 | 42/8048 | 5.89E-06 | 4.78E-06 |
| hsa04940 | Type I diabetes mellitus | 7/93 | 43/8048 | 6.47E-06 | 5.26E-06 |
| hsa04060 | Cytokine-cytokine receptor interaction | 15/93 | 294/8048 | 1.33E-05 | 1.08E-05 |
| hsa05310 | Asthma | 6/93 | 31/8048 | 1.33E-05 | 1.08E-05 |
| hsa05320 | Autoimmune thyroid disease | 7/93 | 53/8048 | 2.32E-05 | 1.89E-05 |
| hsa05322 | Systemic lupus erythematosus | 10/93 | 136/8048 | 3.34E-05 | 2.72E-05 |
| hsa04062 | Chemokine signaling pathway | 10/93 | 189/8048 | 0.000567 | 0.000461 |
| hsa05321 | Inflammatory bowel disease | 6/93 | 65/8048 | 0.00087 | 0.000707 |

Supplementary Table 7. The list of 48 potential immune-related genes.

| ABCA6 | ABI3BP | ADAMDEC1 | ADH1B | ALOX5AP | AQP4 |
| --- | --- | --- | --- | --- | --- |
| AQP9 | ARHGEF6 | C16orf89 | C1orf162 | C7 | CCDC69 |
| CD52 | CHRDL1 | CP | CXCL13 | CYP4B1 | DMBT1 |
| DPT | FBP1 | FCN3 | FDCSP | FLRT3 | GIMAP4 |
| GMFG | HPGD | HPGDS | IL7R | LYVE1 | MFAP4 |
| MMP7 | MMRN1 | NAPSA | NDNF | NEXN | OLR1 |
| PBK | PEBP4 | PECAM1 | PGC | PLA2G1B | RASSF2 |
| SCGB3A1 | SFTPB | SLC6A14 | SPINK1 | TREM1 | VGLL3 |

Supplementary Table 8. The distribution of TICs between the tumor and control tissue samples.

| **Immune cell** | **Median Control/Tumor** | **Mann-Whitney U** | **P value** |  |
| --- | --- | --- | --- | --- |
| **B cells naive** | 0.01161/0.01617 | 49785 | 0.0126 |  |
| **B cells memory** | 0/0 | 51352 | 0.0113 |  |
| **Plasma cells** | 0.01754/0.07245 | 23708 | < 0.0001 |  |
| **T cells CD8** | 0.1029/0.1018 | 56149 | 0.9611 |  |
| **T cells CD4 naive** | 0/0 | 47214 | 0.476 |  |
| **T cells CD4 memory resting** | 0.07665/0.04030 | 42521 | < 0.0001 |  |
| **T cells CD4 memory activated** | 0.02565/0.03236 | 51419 | 0.0622 |  |
| **T cells follicular helper** | 0.01195/0.03476 | 34986 | < 0.0001 |  |
| **T cells regulatory (Tregs)** | 0/0 | 46953 | < 0.0001 |  |
| **T cells gamma delta** | 0.01494/0.01421 | 55931 | 0.8911 |  |
| **NK cells resting** | 0.03487/0 | 30827 | < 0.0001 |  |
| **NK cells activated** | 0.002572/0 | 49860 | 0.0072 |  |
| **Monocytes** | 0.06033/0 | 13335 | < 0.0001 |  |
| **Macrophages M0** | 0.06334/0.09865 | 42765 | < 0.0001 |  |
| **Macrophages M1** | 0.03805/0.09542 | 27229 | < 0.0001 |  |
| **Macrophages M2** | 0.07591/0.07513 | 55843 | 0.8684 |  |
| **Dendritic cells resting** | 0.04933/0.1157 | 34667 | < 0.0001 |  |
| **Dendritic cells activated** | 0.01754/0 | 33543 | < 0.0001 |  |
| **Mast cells resting** | 0.05982/0.003337 | 34555 | < 0.0001 |  |
| **Mast cells activated** | 0.007436/0.01741 | 55334 | 0.7092 |  |
| **Eosinophils** | 0.04287/0 | 17886 | < 0.0001 |  |
| **Neutrophils** | 0.04381/0.006903 | 25208 | < 0.0001 |  |

Supplementary Table 9. 16 potential immune-related genes showed similar regression trends for someone of TICs in both tumor and control samples.

|  |  |  | B cells memory | |  |
| --- | --- | --- | --- | --- | --- |
|  |  | Coefficient | Std. Error | t-Statistic | Prob. |
|  | **Control** | 0.024325 | 0.005145 | 4.728198 | 0 |
|  | **Tumor** | 0.011621 | 0.003827 | 3.036493 | 0.0025 |
| **ALOX5AP** |  |  | T cells follicular helper | |  |
|  |  | Coefficient | Std. Error | t-Statistic | Prob. |
|  | **Control** | -0.010344 | 0.003685 | -2.806955 | 0.0056 |
|  | **Tumor** | -0.006339 | 0.003346 | -1.894756 | 0.0588 |
|  |  |  | Neutrophils | |  |
|  |  | Coefficient | Std. Error | t-Statistic | Prob. |
| **AQP9** | **Control** | 0.031093 | 0.004421 | 7.033038 | 0 |
|  | **Tumor** | 0.015034 | 0.002285 | 6.579645 | 0 |
|  |  |  | Neutrophils | |  |
|  |  | Coefficient | Std. Error | t-Statistic | Prob. |
| **ARHGEF6** | **Control** | -0.030754 | 0.00951 | -3.233805 | 0.0015 |
|  | **Tumor** | -0.011397 | 0.004024 | -2.832402 | 0.0048 |
|  |  |  | Plasma cells |  |  |
|  |  | Coefficient | Std. Error | t-Statistic | Prob. |
| **C1ORF162** | **Control** | -0.019464 | 0.011191 | -1.739199 | 0.0838 |
|  | **Tumor** | -0.030061 | 0.008425 | -3.568214 | 0.0004 |
|  |  |  | T cells CD4 memory resting | |  |
|  |  | Coefficient | Std. Error | t-Statistic | Prob. |
|  | **Control** | -0.038685 | 0.010124 | -3.821118 | 0.0002 |
|  | **Tumor** | -0.013934 | 0.006503 | -2.14264 | 0.0327 |
|  |  |  | Neutrophils | |  |
|  |  | Coefficient | Std. Error | t-Statistic | Prob. |
| **CD52** | **Control** | -0.010179 | 0.005643 | -1.803719 | 0.073 |
|  | **Tumor** | -0.011019 | 0.003086 | -3.570994 | 0.0004 |
|  |  |  | B cells memory | |  |
|  |  | Coefficient | Std. Error | t-Statistic | Prob. |
|  | **Control** | 0.008228 | 0.004337 | 1.897155 | 0.0595 |
|  | **Tumor** | 0.012488 | 0.004438 | 2.81385 | 0.0051 |
|  |  |  | T cells follicular helper | |  |
|  |  | Coefficient | Std. Error | t-Statistic | Prob. |
|  | **Control** | 0.004394 | 0.00133 | 3.303914 | 0.0012 |
| **CXCL13** | **Tumor** | 0.008564 | 0.001429 | 5.990779 | 0 |
|  |  |  | Macrophages M0 |  |  |
|  |  | Coefficient | Std. Error | t-Statistic | Prob. |
|  | **Control** | -0.008417 | 0.004932 | -1.706727 | 0.0897 |
|  | **Tumor** | -0.013421 | 0.003761 | -3.568185 | 0.0004 |
|  |  |  | Macrophages M0 | |  |
|  |  | Coefficient | Std. Error | t-Statistic | Prob. |
| **FBP1** | **Control** | 0.032158 | 0.010427 | 3.084241 | 0.0024 |
|  | **Tumor** | 0.016238 | 0.00685 | 2.370603 | 0.0182 |
|  |  |  | B cells memory | |  |
|  |  | Coefficient | Std. Error | t-Statistic | Prob. |
| **FDCSP** | **Control** | 0.009921 | 0.002662 | 3.72653 | 0.0003 |
|  | **Tumor** | 0.004103 | 0.001487 | 2.759651 | 0.006 |
|  |  |  | Neutrophils | |  |
|  |  | Coefficient | Std. Error | t-Statistic | Prob. |
| **GMFG** | **Control** | 0.041064 | 0.009293 | 4.418842 | 0 |
|  | **Tumor** | 0.012634 | 0.005028 | 2.512795 | 0.0123 |
|  |  |  | Plasma cells | |  |
|  |  | Coefficient | Std. Error | t-Statistic | Prob. |
| **HPGD** | **Control** | -0.011429 | 0.005256 | -2.17442 | 0.031 |
|  | **Tumor** | -0.004656 | 0.002508 | -1.856592 | 0.064 |
|  |  |  | NK cells resting | |  |
|  |  | Coefficient | Std. Error | t-Statistic | Prob. |
|  | **Control** | -0.013496 | 0.004605 | -2.930698 | 0.0038 |
|  | **Tumor** | -0.003827 | 0.001402 | -2.730239 | 0.0066 |
| **HPGDS** |  |  | Monocytes |  |  |
|  |  | Coefficient | Std. Error | t-Statistic | Prob. |
|  | **Control** | -0.011701 | 0.005365 | -2.180827 | 0.0305 |
|  | **Tumor** | -0.003106 | 0.001362 | -2.279421 | 0.0231 |
|  |  |  | Neutrophils | |  |
|  |  | Coefficient | Std. Error | t-Statistic | Prob. |
|  | **Control** | -0.009045 | 0.004118 | -2.196343 | 0.0294 |
|  | **Tumor** | -0.005224 | 0.002011 | -2.59797 | 0.0097 |
|  |  |  | T cells CD4 memory resting | |  |
|  |  | Coefficient | Std. Error | t-Statistic | Prob. |
|  | **Control** | 0.019018 | 0.007388 | 2.574097 | 0.0109 |
| **IL7R** | **Tumor** | 0.017794 | 0.004238 | 4.198897 | 0 |
|  |  |  | T cells follicular helper | |  |
|  |  | Coefficient | Std. Error | t-Statistic | Prob. |
|  | **Control** | -0.004407 | 0.002267 | -1.94377 | 0.0535 |
|  | **Tumor** | -0.00985 | 0.002528 | -3.896348 | 0.0001 |
|  |  |  | NK cells resting | |  |
|  |  | Coefficient | Std. Error | t-Statistic | Prob. |
|  | **Control** | 0.010757 | 0.0043 | 2.501732 | 0.0133 |
|  | **Tumor** | 0.003718 | 0.001273 | 2.920238 | 0.0037 |
|  |  |  | NK cells resting | |  |
|  |  | Coefficient | Std. Error | t-Statistic | Prob. |
|  | **Control** | -0.010358 | 0.005155 | -2.009318 | 0.0461 |
|  | **Tumor** | -0.002651 | 0.001156 | -2.291893 | 0.0224 |
| **OLR1** |  |  | Eosinophils | |  |
|  |  | Coefficient | Std. Error | t-Statistic | Prob. |
|  | **Control** | 0.010782 | 0.004884 | 2.207556 | 0.0286 |
|  | **Tumor** | 0.002347 | 0.00105 | 2.235219 | 0.0259 |
|  |  |  | Macrophages M1 | |  |
|  |  | Coefficient | Std. Error | t-Statistic | Prob. |
| **LYVE1** | **Control** | -0.007448 | 0.004088 | -1.821848 | 0.0702 |
|  | **Tumor** | -0.00916 | 0.003409 | -2.68679 | 0.0075 |
|  |  |  | Plasma cells | |  |
|  |  | Coefficient | Std. Error | t-Statistic | Prob. |
| **PECAM1** | **Control** | 0.027333 | 0.011983 | 2.280902 | 0.0238 |
|  | **Tumor** | 0.015644 | 0.008754 | 1.787094 | 0.0746 |
|  |  |  | Dendritic cells activated | |  |
|  |  | Coefficient | Std. Error | t-Statistic | Prob. |
| **SLC6A14** | **Control** | 0.005536 | 0.002595 | 2.133314 | 0.0343 |
|  | **Tumor** | 0.001474 | 0.000785 | 1.878642 | 0.0609 |

Supplementary Table 10. 9 potential immune-related genes showed opposite regression trends for someone of TICs in tumor and control samples.

|  |  |  | Dendritic cells activated | |  |
| --- | --- | --- | --- | --- | --- |
|  |  | Coefficient | Std. Error | t-Statistic | Prob. |
| ADH1B | Control | -0.041043 | 0.012115 | -3.38777 | 0.0009 |
|  | Tumor | 0.026522 | 0.008173 | 3.245257 | 0.0013 |
|  |  |  | Neutrophils | |  |
|  |  | Coefficient | Std. Error | t-Statistic | Prob. |
| CHRDL1 | Control | 0.023529 | 0.006802 | 3.459156 | 0.0007 |
|  | Tumor | -0.0028 | 0.001687 | -1.659812 | 0.0977 |
|  |  |  | NK cells resting | |  |
|  |  | Coefficient | Std. Error | t-Statistic | Prob. |
| DMBT1 | Control | -0.006428 | 0.003287 | -1.95554 | 0.0521 |
|  | Tumor | 0.001647 | 0.000785 | 2.096787 | 0.0366 |
|  |  |  | Macrophages M0 | |  |
|  |  | Coefficient | Std. Error | t-Statistic | Prob. |
| MMP7 | Control | -0.008359 | 0.004883 | -1.711917 | 0.0887 |
|  | Tumor | 0.007998 | 0.003416 | 2.341198 | 0.0197 |
|  |  |  | Mast cells resting | |  |
|  |  | Coefficient | Std. Error | t-Statistic | Prob. |
| OLR1 | Control | 0.031339 | 0.009168 | 3.418186 | 0.0008 |
|  | Tumor | -0.004243 | 0.00257 | -1.650875 | 0.0995 |
|  |  |  | Eosinophils | |  |
|  |  | Coefficient | Std. Error | t-Statistic | Prob. |
|  | Control | -0.008239 | 0.004277 | -1.926442 | 0.0557 |
|  | Tumor | 0.001595 | 0.000859 | 1.856682 | 0.064 |
| PBK |  |  | Plasma cells | |  |
|  |  | Coefficient | Std. Error | t-Statistic | Prob. |
|  | Control | 0.013753 | 0.005882 | 2.338086 | 0.0205 |
|  | Tumor | -0.005708 | 0.003272 | -1.744314 | 0.0818 |
|  |  |  | NK cells resting | |  |
|  |  | Coefficient | Std. Error | t-Statistic | Prob. |
| PLA2G1B | Control | 0.007589 | 0.004077 | 1.861538 | 0.0644 |
|  | Tumor | -0.001904 | 0.001043 | -1.826333 | 0.0685 |
|  |  |  | B cells naive | |  |
|  |  | Coefficient | Std. Error | t-Statistic | Prob. |
| SCGB3A1 | Control | -0.00351 | 0.001394 | -2.517626 | 0.0127 |
|  | Tumor | 0.001362 | 0.000825 | 1.649579 | 0.0997 |
|  |  |  | Macrophages M0 | |  |
|  |  | Coefficient | Std. Error | t-Statistic | Prob. |
| TREM1 | Control | -2.74E-02 | 0.009435 | -2.904212 | 0.0042 |
|  | Tumor | 0.013968 | 0.004828 | 2.893232 | 0.004 |

Supplementary Table 11. The correlation between 9 differential immune-related genes and 4 known biomarkers.

| Gene symbol | PD-L1 | | CD8A | | GZMB | | NOS2 | |
| --- | --- | --- | --- | --- | --- | --- | --- | --- |
|  | r value | p value | r value | p value | r value | p value | r value | p value |
| ADH1B | 0.0787 | 0.157 | 0.2826 | < 0.0001 | 0.187 | 0.0007 | -0.3384 | < 0.0001 |
| CHRDL1 | 0.1058 | 0.0568 | 0.3047 | < 0.0001 | 0.2297 | < 0.0001 | -0.2581 | < 0.0001 |
| DMBT1 | 0.2206 | < 0.0001 | 0.2719 | < 0.0001 | 0.2907 | < 0.0001 | -0.1633 | 0.0032 |
| MMP7 | 0.3547 | < 0.0001 | 0.3046 | < 0.0001 | 0.3822 | < 0.0001 | 0.0313 | 0.5745 |
| **OLR1** | **0.4275** | **< 0.0001** | **0.4028** | **< 0.0001** | **0.4972** | **< 0.0001** | **-0.4169** | **< 0.0001** |
| PBK | 0.0047 | 0.9326 | -0.0441 | 0.4286 | 0.0658 | 0.2366 | 0.3342 | < 0.0001 |
| PLA2G1B | 0.0411 | 0.4602 | 0.0847 | 0.1275 | -0.0006 | 0.9909 | -0.3841 | < 0.0001 |
| SCGB3A1 | 0.1523 | 0.0059 | 0.267 | < 0.0001 | 0.2121 | 0.0001 | -0.1327 | 0.0167 |
| TREM1 | 0.2849 | < 0.0001 | 0.3117 | < 0.0001 | 0.3925 | < 0.0001 | -0.2236 | < 0.0001 |
| PD-L1 | 1 | - | 0.5988 | < 0.0001 | 0.6412 | < 0.0001 | -0.1747 | 0.0016 |
| CD8A | 0.5988 | < 0.0001 | 1 | - | 0.7853 | < 0.0001 | -0.0746 | 0.18 |
| GZMB | 0.6412 | < 0.0001 | 0.7853 | < 0.0001 | 1 | - | -0.1941 | 0.0004 |
| NOS2 | -0.1747 | 0.0016 | -0.0746 | 0.18 | -0.1941 | 0.0004 | 1 | - |

Supplementary Table 12. 4 known biomarker showed more positive expression for immunotherapy in Top 50% OLR expression group patients.

|  | | OLR1 | | chi-square value | p value |
| --- | --- | --- | --- | --- | --- |
|  |  | Bottom 50% | Top 50% |  |  |
| PD-L1 | Bottom 50% | 120 | 59 | 43.952 | P<0.0001 |
|  | Top 50% | 43 | 103 |  |  |
| CD8A | Bottom 50% | 104 | 58 | 24.374 | P<0.0001 |
|  | Top 50% | 59 | 104 |  |  |
| GZMB | Bottom 50% | 102 | 50 | 31.56 | P<0.0001 |
|  | Top 50% | 61 | 112 |  |  |
| NOS2 | Bottom 50% | 63 | 105 | 21.239 | P<0.0001 |
|  | Top 50% | 100 | 57 |  |  |

Supplementary Table 13. The correlation between OLR1 expression and all indicators of TIDE prediction.

| **Gene symbol** | **OLR1** | | |
| --- | --- | --- | --- |
|  | **r value** | **p value** | **95% confidence interval** |
| Tide prediction score | -0.1154 | 0.253 | -0.3049 to 0.08297 |
| IFNG signatures expression | 0.3968 | < 0.0001 | 0.2173 to 0.5504 |
| MSI score (TCGA Prediction) | 0.5706 | < 0.0001 | 0.4213 to 0.6897 |
| T cell-inflamed signature Merck18 | 0.757 | < 0.0001 | 0.6585 to 0.8300 |
| PD-L1 expression | -0.2632 | 0.0082 | -0.4370 to -0.07035 |
| Average of CD8A and CD8B | 0.7407 | < 0.0001 | 0.6369 to 0.8181 |
| T cell dysfunction potential of tumor | 0.2729 | 0.0060 | 0.08072 to 0.4454 |
| T cell exclusion potential of tumor | -0.5008 | < 0.0001 | -0.6348 to -0.3376 |
